# Areca nut-induced metabolic reprogramming and M2 differentiation promote OPMD malignant transformation

**DOI:** 10.1186/s13046-024-03163-z

**Published:** 2024-08-20

**Authors:** Shyng-Shiou F. Yuan, Leong-Perng Chan, Hieu D. H. Nguyen, Chang-Wei Su, Yuk-Kwan Chen, Jeff Yi-Fu Chen, Shigetaka Shimodaira, Stephen Chu‐Sung Hu, Steven Lo, Yen-Yun Wang

**Affiliations:** 1 Graduate Institute of Medicine, College of Medicine, Kaohsiung Medical University, Kaohsiung, Taiwan; 2 Translational Research Center, Kaohsiung Medical University Hospital, Kaohsiung, 807 Taiwan; 3 Department of Obstetrics and Gynecology, Kaohsiung Medical University Hospital, Kaohsiung, Taiwan; 4 Department of Medical Research, Kaohsiung Medical University Hospital, Kaohsiung, 807 Taiwan; 5 Drug Development and Value Creation Research Center, Kaohsiung Medical University, Kaohsiung, Taiwan; 6 Department of Biological Science and Technology, Institute of Molecular Medicine and Bioengineering, Center for Intelligent Drug Systems and Smart Biodevices (IDS2B), National Yang Ming Chiao Tung University, 75 Bo-Ai Street, Hsinchu, Taiwan; 7 Cohort Research Center, Kaohsiung Medical University, Kaohsiung, Taiwan; 8 Faculty of Medicine, College of Medicine, Kaohsiung Medical University, Kaohsiung, Taiwan; 9 Department of Otorhinolaryngology-Head and Neck Surgery, Kaohsiung Municipal Ta-Tung Hospital and Kaohsiung Medical University Hospital, Kaohsiung, Taiwan; 10 School of Dentistry, College of Dental Medicine, Kaohsiung Medical University, No.100, Shih-Chuan 1st Road, Sanmin Dist., Kaohsiung, 80708 Taiwan; 11 Division of Oral and Maxillofacial Surgery, Kaohsiung Medical University Hospital, Kaohsiung, Taiwan; 12 Division of Oral Pathology & Maxillofacial Radiology, Kaohsiung Medical University Hospital, Kaohsiung, Taiwan; 13 Department of Biotechnology, Kaohsiung Medical University, Kaohsiung, Taiwan; 14 Department of Regenerative Medicine, Kanazawa Medical University, Kahoku, Ishikawa 920-0293 Japan; 15 Center for Regenerative Medicine, Kanazawa Medical University Hospital, Kahoku, Ishikawa 920-0293 Japan; 16 Division of Stem Cell Medicine, Department of Advanced Medicine, Medical Research Institute, Kanazawa Medical University, Kahoku, Ishikawa 920-0293 Japan; 17 Department of Transfusion Medicine and Cell Processing, Tokyo Women’s Medical University, Shinjuku, Tokyo, 162-8666 Japan; 18 Department of Dermatology, College of Medicine, Kaohsiung Medical University, Kaohsiung, 807 Taiwan; 19 Department of Dermatology, Kaohsiung Medical University Hospital, Kaohsiung, 807 Taiwan; 20 Canniesburn Regional Plastic Surgery and Burns Unit, Glasgow, G4 0SF UK; 21 College of Medical, Veterinary and Life Sciences, University of Glasgow, Glasgow, G12 8QQ UK

**Keywords:** Areca nut, Macrophage, OPMD, OSCC, Mitochondrial metabolism

## Abstract

**Background:**

Betel quid and its major ingredient, areca nut, are recognized by IARC as major risk factors in oral cancer development. Areca nut extract (ANE) exposure has been linked to OPMD progression and malignant transformation to OSCC. However, the detailed mechanism through which ANE acts on other cell types in the oral microenvironment to promote oral carcinogenesis remains elusive.

**Methods:**

Immunoprofiling of macrophages associated with OPMD and OSCC was carried out by immunohistochemical and immunofluorescence staining. Phosphokinase and cytokine arrays and western blotting were performed to determine the underlying mechanisms. Transwell assays were used to evaluate the migration-promoting effect of ANE. Hamster model was finally applied to confirm the in vivo effect of ANE.

**Results:**

We reported that M2 macrophages positively correlated with oral cancer progression. ANE induced M2 macrophage differentiation, CREB phosphorylation and VCAM-1 secretion and increased mitochondrial metabolism. Conditioned medium and VCAM-1 from ANE-treated macrophages promoted migration and mesenchymal phenotypes in oral precancer cells. In vivo studies showed that ANE enhanced M2 polarization and related signaling pathways in the oral buccal tissues of hamsters.

**Conclusion:**

Our study provides novel mechanisms for areca nut-induced oral carcinogenesis, demonstrating that areca nut promotes M2 macrophage differentiation and secretion of oncogenic cytokines that critically activate malignant transformation of oral premalignant cells.

**Supplementary Information:**

The online version contains supplementary material available at 10.1186/s13046-024-03163-z.

## Background

Macrophages are innate immune cells present in almost all tissues with diverse functions in response to environmental challenges [1]. Accumulating evidence indicates that infiltrating macrophages in the tumor microenvironment, known as tumor-associated macrophages (TAMs), play a critical role in various types of cancers, such as ovarian [2], breast [3], gastric [4], and oral cancer [5]. Infiltrating macrophages in the tumor microenvironment show preferential expression of particular cell surface marker proteins, including CD163, CD206, and CD204, and enhance cancer progression via secretion of numerous cytokines, growth factors, and inflammatory substrates [6–9]. These macrophage markers are referred to as M2-polarized macrophages, differentiating them from M1-polarized macrophages, which have reduced expression of these cell surface markers and demonstrate anticancer activities [10]. In contrast, M2 macrophages may be involved in the early phase of oral cancer development, with M2 macrophage infiltration increased in oral premalignant lesions [11, 12] and associated with advanced pathological grade and poor prognosis in oral cancer patients [9, 13].

Areca nut has been identified as a causative factor of betel quid chewing-associated oral carcinogenesis [14, 15], consisting of four major components, including arecoline, arecaidine, guvacoline, and guvacine [14], among which arecoline is the main compound present in the saliva during and post-areca nut chewing [14, 16]. Areca nut extract or arecoline may cause cytopathological changes and accumulation of DNA damage in oral epithelial cells [17] and mucosal fibroblasts [18]. Additionally, ANE promotes the transformation of oral potentially malignant disorders (OPMD) to oral squamous cell carcinoma (OSCC) via activation of oral immune cells, such as macrophages, in the oral tissue microenvironment [19, 20]. Furthermore, ANE-induced cytotoxicity in oral premalignant cells has been reported to be associated with mitochondrial abnormalities, resulting in the accumulation of excessive amounts of intracellular reactive oxygen species [21].

According to the latest Annual Report of Ministry of Health and Welfare, Taiwan, oral cancer ranks as the 4th most prevalent cancer type in the Taiwanese male population and the 5th leading cause of cancer death in both genders [22]. The International Agency for Research on Cancer (IARC) database has projected that the global annual incidence of oral cancer may increase from 377,713 cases in 2020 to 553,481 cases in 2040, indicating a rise of 46.5% [23]. In this regard, it is therefore vital to seek novel early interventions that prevent the progression of OPMD to oral cancer [24].

ANE exposure has been linked to OPMD progression and subsequent malignant transformation to OSCC. However, the detailed mechanism through which ANE acts on other cell types in the oral microenvironment to promote oral carcinogenesis remains elusive. Therefore, in this study, we aimed to study how ANE affects microenvironmental macrophages to influence the malignant transformation of oral premalignant cells. Elucidation of this complex pathogenic process may inspire the development of targeted therapies for the prevention and early intervention of ANE-induced oral malignant transformation.

## Methods

### Cell culture

The human leukemia monocytic cell line THP-1 was cultured in RPMI 1640 medium (Thermo Fisher Scientific), while the oral premalignant cell line DOK was maintained in DMEM/F12 medium (Thermo Fisher Scientific). All cell lines were maintained in their basic cell culture medium supplemented with 10% fetal bovine serum (Biological Industries), 1% glutamine (Merck), and 1 × penicillin/streptomycin/amphotericin B solution (100 × ; Merck). The cells were placed in a humidified incubator with 5% CO_2_ at 37 °C.

### Peripheral blood mononuclear cell isolation

Peripheral blood mononuclear cells (PBMCs) were isolated from the whole blood of healthy donors by density gradient centrifugation using Histopaque (Sigma). The donors had agreed and signed an informed consent form approved by the Institutional Review Board (IRB Approval no. KMUHIRB-F(I)-20180069) of Kaohsiung Medical University Hospital (Kaohsiung, Taiwan). Briefly, blood samples were diluted with the same amount of phosphate buffered saline (PBS) and subjected to centrifugation at 400 × g for 40 min at 25 °C. The white layer representing PBMCs was aspirated out gently and transferred into a 15 mL sterile centrifuge tube. After the addition of an equal amount of PBS, the tube was centrifuged at 400 × g for 10 min, and the supernatant containing platelets was discarded. The cell pellet was gently mixed with RPMI 1640 complete medium (Thermo Fisher Scientific) and incubated in a culture plate for 3 h. At the indicated time points, the supernatant containing lymphocytes was aspirated, and the remaining monocytes were allowed to settle onto the culture plate and maintained in RPMI 1640 complete medium supplemented with 10% fetal bovine serum (Biological Industries), 1% glutamine (Merck), and 1 × penicillin/streptomycin/amphotericin B solution (Merck). The cells were placed in a humidified incubator with 5% CO_2_ at 37 °C for further experiments.

### Conditioned medium culture and drug treatment

THP-1 cells were seeded in 6 cm dishes at a density of 2 × 10^6^, primed with 50 nM PMA for 6 h and refreshed with 5 mL culture medium. The cells were then treated with ANE (0.4 mg/mL for THP-1 cells and DOK cells; 0.1 µg/ml for PBMCs) or arecoline (0.1 mM and 0.3 mM) for 48 h. For functional studies, the CREB inhibitor 666–15 (80 nM, TOCRIS), the CREB inhibitor KG-501 (10 μM, MedchemExpress) [25, 26] which is a gift from lab of Prof. Long-Sen Chang [27], the VCAM-1 inhibitor δ-tocotrienol (20 µM, Cayman) was added to the culture media 30 min before adding ANE. Conditioned media were collected at 48 h after ANE treatment.

### XTT cell viability assay

Cells were incubated for designated periods, and then cell viability/cell proliferation was analyzed using the XTT assay (Sigma‒Aldrich, St. Louis, MO). In brief, the media were replaced with 150 µl XTT solution (50 µg XTT and 0.4 µg phenazine methosulfate in 150 µl cell culture medium) and incubated at 37 °C for 2 h, followed by measuring OD at 470 nm with subtraction of the background at OD 660 nm.

### Annexin V staining

To detect apoptotic cells after ANE or arecoline (FL-31593-250MG, Sigma‒Aldrich, St. Louis, MO) treatment, a FITC Annexin V Apoptosis Detection Kit I (BD Biosciences, San Jose, CA) was applied. THP-1 cells were seeded on 6 cm plates at a density of 1 × 10^6^ cells per plate, and cells were collected after ANE or arecoline treatment for 48 h. After washing three times with precooled 1 × PBS, the cells were stained with Annexin V working solution (5 µL of Annexin V-FITC reagent and 5 µL of propidium iodide (PI) solution in 100 µL of 1 × Annexin V binding buffer) for 15 min at room temperature. Cells were analyzed with a Cytomics FC 500 flow cytometer (Beckman Coulter, Brea, CA). The fluorescence signals of Annexin V-FITC and PI were evaluated by FL-1 and FL-3 channels, respectively. The data were analyzed with MFA32 software (Beckman Coulter, Brea, CA).

### Flow cytometric assay of surface markers

THP-1 cells were primed with 50 nM PMA for 6 h, followed by ANE treatment for 48 h. After washing three times with flow cytometer buffer, the cells were fixed with 4% paraformaldehyde for 30 min and then stained with CD163 (ab182422, Abcam, Cambridge, UK), CD206 (bs4727R, Bioss, MA), or HLA-DR (ab175085, Abcam, Cambridge, UK) antibodies for 30 min. The fluorescence intensity was evaluated with a Cytomics FC 500 flow cytometer (Beckman Coulter, Brea, CA), and data were analyzed with FlowJo software version 10 (Tree Star Inc., San Carlos, CA).

### Phospho-kinase array

A human phospho-kinase antibody array (ARY003C, R&D Systems) was applied to discover the potential signaling pathways mediating the effects of ANE in THP-1 cells. The specific sites of phosphorylation in 43 kinases were determined by using THP-1 cell lysates according to the manufacturer’s instructions.

### Cytokine array

THP-1 cells were treated with ANE (0.4 mg/ml) for 48 h, and the conditioned media were applied to the cytokine array membranes (Proteome Profiler Human XL Cytokine Array Kit, ARY002B, R&D systems). The spot signals were developed according to the manufacturer’s instructions.

### Cell secretome

To evaluate the cell secretome, THP-1 cells were treated with ANE for 48 h, and the conditioned media were filtered and frozen at -80 °C until further analysis. The concentration of the secretome was quantified using the appropriate DuoSet® Immunoassay Development kits (R&D systems) according to the manufacturer’s instructions.

### Analysis of ANE components by LC/MS

ANE components were analyzed using a TSQ Quantum Ultra mass spectrometer (Thermo Scientific, Waltham, MA). A C18 column with 150 × 0.5 mm i.d., 5 μm (Agilent, Santa Clara, CA) was used to separate the components. The mobile phase consisted of 0.1% formic acid in water (solvent A) and 0.1% formic acid in methanol (solvent B), with a flow rate of 15 μL/min. The injection volume was 3 µL with a column oven at 35 °C. The mass spectrometer with an electrospray ionization source was set in both negative and positive ionization modes. The capillary temperature was maintained at 250 °C, the source voltage and spray voltage were set at 3500 V, and the collision gas (Ar) pressure was set at 1.4 mTorr. The scan width was set at 0.4 Da with a scan time of 0.10 s.

### Transwell migration assay

DOK cells were treated with the indicated conditioned medium (CM) for 48 h, and cells were harvested and seeded in 8 μM pore size transwell inserts at a density of 5 × 10^4^ in 100 μL serum-free medium. The insert was carefully placed into a single well of a 24-well plate containing 500 μL serum-containing medium and incubated for 72 h. After incubation, the cells remaining on the upper chamber were removed using cotton swabs, while the cells that migrated into the lower chamber were stained with crystal violet solution for 15 min. The percentage of cell migration was calculated using ImageJ software.

### Western blotting

Immunoblotting was performed as described previously [28]. SDS‒PAGE was performed, and proteins were transferred to PVDF membranes for further analysis. The chemiluminescence signal was captured by a Gel DocTM XR + Gel documentation system (Bio-Rad, Hercules, CA). The following primary antibodies were used: recombinant anti-human CD163 (ab182422, Abcam, Cambridge, UK), rabbit anti-human VCAM-1 (#13,662, Cell Signaling Technology, Massachusetts), rabbit anti-human p-CREB (GTX61045, Genetex, Irvine, CA), rabbit anti-human CREB (GTX112846, Genetex, Irvine, CA), rabbit anti-human EGFR (phosphor Tyr1086, GTX133599, Genetex, Irvine, CA), rabbit anti-human EGFR (GTX100448, Genetex, Irvine, CA), recombinant anti-human phospho-c-Jun (S63, MAB8930, R&D Systems, MN), mouse anti-human c-Jun (sc-166544, Santa Cruz, Texas), rabbit anti-human N-Cadherin (#4061, Cell Signaling Technology, Massachusetts), rabbit anti-human vimentin (GTX100619, Genetex, Irvine, CA), rabbit anti-human claudin-1 (#4933, Cell Signaling Technology, Massachusetts), rabbit anti-human ZO-1 (GTX108627, Genetex, Irvine, CA), mouse anti-human beta-actin (A5441, Sigma-Aldrich, St. Louis, MO). The secondary antibodies used in this study were goat anti-mouse IgG antibody (GTX213111-01, GeneTex, Irvine, CA), goat anti-rabbit IgG antibody (GTX213110-01, GeneTex, Irvine, CA), and rabbit anti-goat IgG antibody (GTX228416-01, GeneTex, Irvine, CA).

### Co-immunoprecipitation (Co-IP)

A co-IP assay was performed with a Pierce Crosslink Magnetic IP/Co-IP kit (88,805, Thermo Fisher Scientific) following the manufacturer’s instructions. Briefly, 5 µg of primary antibodies were covalently cross-linked with 25 µl of protein A/G magnetic beads. Protein extraction was performed using Pierce IP lysis/wash buffer and protease and phosphatase inhibitor cocktail. A portion of each sample was used as input, while equal amounts (0.5 mg) of each protein extract were incubated with protein A/G magnetic beads cross-linked with primary antibodies overnight at 4 °C. The beads were then washed three times with 1 × modified coupling buffer to remove unbound proteins, and the bound proteins were then eluted from the antibody-crosslinking beads by a low-pH elution buffer. The elute was mixed with neutralization buffer to neutralize the low pH. Immunoprecipitates were analyzed using Western blotting. The primary antibodies used in this experiment included IGTα4 (8840, Cell Signaling Technology, Massachusetts) and ubiquitin (GTX19247, GeneTex, Irvine, CA).

### Measurement of the oxygen consumption rate (OCR) and extracellular acidification rate (ECAR) using the CLARIOstar system

The basal oxidative phosphorylation (determined by OCR) and glycolysis (determined by ECAR) in human monocytic THP-1 cells and oral premalignant DOK cells were measured using a CLARIOstar Plus plate reader (BMG LABTECH, Germany). To analyze OCR, the cells were seeded in 96-well culture plates (black wall with clear flat bottom) at a density of 8 × 10^4^ cells/well in 200 µl media and cultured overnight. The media were refreshed, and after the addition of the extracellular O_2_ consumption reagent (ab197243, Abcam, Cambridge, UK), the wells were promptly sealed with prewarmed mineral oil. The extracellular O_2_ consumption signal was measured in the CLARIOstar Plus plate reader at 1.5 min intervals for 120 min at Ex/Em = 360/650 nm. To measure the ECAR rate, the cells were seeded in 96-well plates (black wall with clear flat bottom) at a density of 8 × 10^4^ cells/well in 200 µl media and cultured overnight. After purging CO_2_ in a CO_2_-free incubator at 37 °C with 95% humidity for 3 h, the media were replaced with respiration buffer containing glycolysis assay reagent (ab197244, Abcam, Cambridge, UK). The glycolysis signal (lifetime signal) was measured in the CLARIOstar Plus plate reader at 1.5 min intervals for over 120 min at Ex/Em = 380/615 nm.

### Analysis of mitochondrial function by Agilent Seahorse XF analyzer

To analyze the key parameters of functional mitochondria, an Agilent Seahorse XF analyzer (Agilent Technologies., Wilmington, DE) was applied following the detailed procedure provided by the manufacturer. There were four modulators (Seahorse XF Cell Mito Stress Test Kit) for studying the complexes of the electron transport chain (ETC) in mitochondria, namely, oligomycin, FCCP (carbonyl cyanide-4 (trifluoromethoxy) phenylhydrazone), rotenone and antimycin (all reagents from Sigma‒Aldrich), which were injected into the built-in injection ports of XF sensor cartridges. Oligomycin, an ATP synthase (ETC complex V) inhibitor, was injected first to slow electron flow through the ETC and then decrease mitochondrial respiration (OCR) and cellular ATP production. Then, FCCP, an uncoupling agent that provides an uninhibited electron flow via the ETC to consume oxygen, was injected. Finally, the complex I inhibitor rotenone and the complex III inhibitor antimycin A were injected.

### Patient specimen collection

All normal oral mucosa, OPMD, and OSCC tissue specimens were collected from Kaohsiung Medical University Hospital (Kaohsiung, Taiwan) after approval by the Institutional Review Board (IRB) of Kaohsiung Medical University Hospital (Approval no. KMUH-IRB-20130300, KMUHIRB-E(I)-20210343, KMUHIRB-E(I)-20190009 and KMUHIRB-F(I)-20220016, and KMUHIRB-F(I)-20180069).

### Hamster buccal pouch model

The hamster buccal pouch model has been widely used in vivo to study oral lesions, including in our previous reports [29, 30]. There were two experimental groups in this study, including (1) double-distilled water (solvent for ANE) alone and (2) ANE. In brief, the buccal pouch region of hamsters was painted daily with ddH_2_O or ANE. At the indicated time points, the hamsters were sacrificed to collect buccal tissue, which was divided equally into 4 parts for further analysis.

### Hematoxylin and eosin (H&E) staining

The tissue sections were immersed in hematoxylin for 5 min and then washed with double-distilled water for 15 min. After quickly dipping in 1% acid alcohol, the tissue sections were washed with double-distilled water for 15 min, immersed in 70% ethanol for 3 min, and then immersed in eosin for 1 min. Next, the tissue sections were washed with double-distilled water for 15 min and dehydrated by immersion in 95% alcohol twice followed by 100% twice, with 2 min for each step. The sections were immersed in xylene for 5 min twice and then mounted with Clearium Mounting Media (3,801,100, Leica, CA).

### Immunohistochemistry

For immunohistochemistry (IHC), oral tissue slides were baked, dewaxed and stained with avidin–biotin complexes following our previous procedures [28–30]. Immunohistochemical staining was performed using a BOND-MAX IHC staining machine (Leica Microsystems). When the staining process was completed, the percentage of positively stained cells on the tissue slide was calculated as one of the following categories: 0 (0–4%), 1 (5–24%), 2 (25–49%), 3 (50–74%), and 4 (75–100%). The intensity of staining was scored as follows: 0 (negative), 1 (weak), 2 (moderate), and 3 (strong). The score was evaluated independently by two experts under the same imaging state.

### Immunofluorescence (IF)

In addition to conventional immunohistochemistry for oral tissue sections with single staining, multifluorescence immunohistochemistry was carried out according to previously described procedures [31, 32], and the percentage of cells positively stained with multifluorescence was calculated as [(number of multifluorescent stained cells)/(number of all cells)] × 100% from five random fields.

### Statistical analysis

All statistical analyses were performed using JMP version 14.0 for Windows (SAS Institute, Cary, NC, USA). The differences among groups were analyzed using either Student’s t test or ANOVA. For in vitro studies, data are presented as the mean ± SD from three independent experiments. The results were considered statistically significant if the *p* value was less than 0.05.

## Results

### M2 macrophages were positively correlated with oral cancer progression

**Fig. 1:**
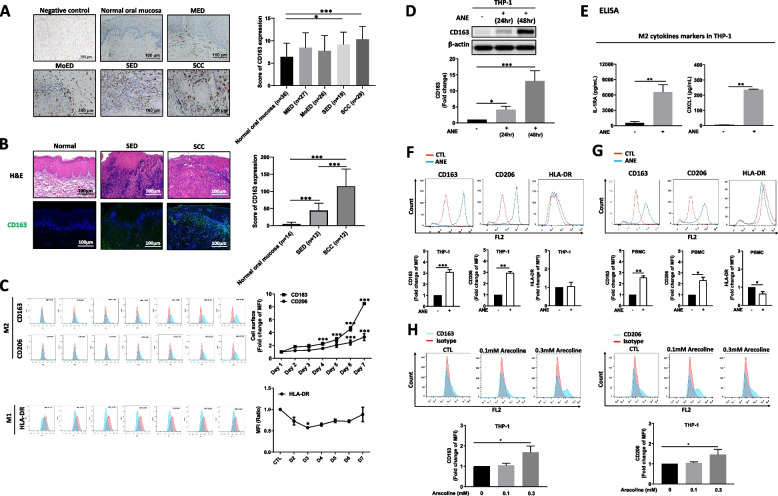
M2 macrophages in the tumor microenvironment were positively correlated with oral lesion progression. **A** The expression of CD163 increased in the oral tissue sections of mild epithelial dysplasia (MED), moderate epithelial dysplasia (MoED), severe epithelial dysplasia (SED), and OSCC patients, scale bar = 100 μm. **B** Fluorescent immunohistochemistry for CD163 expression in oral tissue sections, scale bar = 100 μm. **C** Expression of the M2 cell surface markers CD163 and CD206, and M1 cell surface marker HLA-DR in THP-1 cells. **D** CD163 expression in THP-1 cells at 24 h and 48 h after ANE treatment. **E** Secretion of the M2 cytokines IL-1RA and CXCL1 in THP-1 cells. **F** ANE promoted M2 cell surface markers but not M1 cell surface markers in THP-1 cells after treatment for 48 h. **G** ANE treatment for 48 h promoted the expression of M2 cell surface markers but not M1 cell surface markers in PBMCs. **H** Arecoline promoted M2 cell surface markers in THP-1 cells after treatment for 48 h. Data are presented as the mean ± SD from three independent experiments. *, *p* < 0.05, **, *p* < 0.01, ***, *p* < 0.005

We first investigated whether there was increased infiltration of M2 macrophages in specimens from oral precancer and OSCC patients. Using immunohistochemistry, the expression of CD163 (a protein marker for M2 macrophages) in the oral tissue sections increased gradually from normal oral mucosa to mild, moderate and severe stages of oral epithelial dysplasia (abbreviated as MED, MoED, and SED, respectively) and OSCC (Fig. 1A). This finding was further confirmed using immunofluorescence staining, which showed that CD163 expression was significantly higher in OSCC tissues than in SED and normal oral mucosa tissues (Fig. 1B).

### ANE induced M2 macrophage differentiation in THP-1 cells and PBMCs

Since areca nut is the major carcinogen for oral cancer in South and Southeast Asia [14, 15], we applied an in vitro model to study the effect of areca nut extract (ANE) on macrophage polarization. ANE was prepared according to the procedure established previously [33]. The human monocyte cell line THP-1 was chosen in this research since it is a well-characterized cell model for studying macrophage activities [34]. Cell viability was assessed using Annexin V staining in THP-1 cells to find a suitable concentration of ANE that did not significantly kill THP-1 cells. As shown in Fig. S1A, ANE at a concentration of 0.6 mg/ml led to significant apoptosis after 72 h of treatment compared with 0, 0.2, and 0.4 mg/ml ANE. Notably, THP-1 cells treated with 0.4 mg/ml ANE showed a significant increase in the population of CD163^+^ cells (Fig. S1B). Furthermore, THP-1 cells treated with 0.4 mg/ml ANE for 7 days showed a significant increase in the CD163^+^ cell population (Fig. 1C). Additionally, Western blotting showed that CD163 expression was significantly higher in THP-1 cells after treatment with 0.4 mg/ml ANE for 48 h (Fig. 1D). ELISA analysis was further applied to examine the expression of the M2 cytokines IL-1RA and CXCL1, which were found to be significantly increased in THP-1 cells treated with ANE (Fig. 1E). In addition, ANE treatment of both THP-1 cells and PBMCs resulted in elevated populations of CD163^+^ and CD206^+^ cells (Fig. 1F and G). Arecoline is one of the main components of ANE (Fig. S2B), dose- and time-dependent cytotoxic effects of arecoline were reported in previous studies [35–37]. In this study, we found that 0.3 mM arecoline did not affect cell viability while 0.6 mM arecoline caused more than 90% cell death (Fig. S2A). Additionally, treatment with 0.3 mM arecoline significantly increased the population of CD163^+^ and CD206^+^ cells compared to the untreated group (Fig. 1H).

In this study, we aimed to study how ANE modulates the biological behaviors of oral epithelial cells indirectly through macrophages but not directly. To avoid the possibility that the remaining ANE in THP-1 conditioned medium may directly influence DOK cells, we analyzed the arecoline concentration in conditioned medium using LC/MS. Our results demonstrated that the arecoline concentration in fresh medium was 27,140 ppb (ANE), which dropped to 32 ppb after culture for 2 days (ANE CM) (Fig. S2D), suggesting that the activity of THP-1 conditioned medium on DOK cells was mediated by secretion from THP-1 cells, not ANE itself.

### ANE induced VCAM-1 secretion from THP-1 cells and PBMCs

**Fig. 2:**
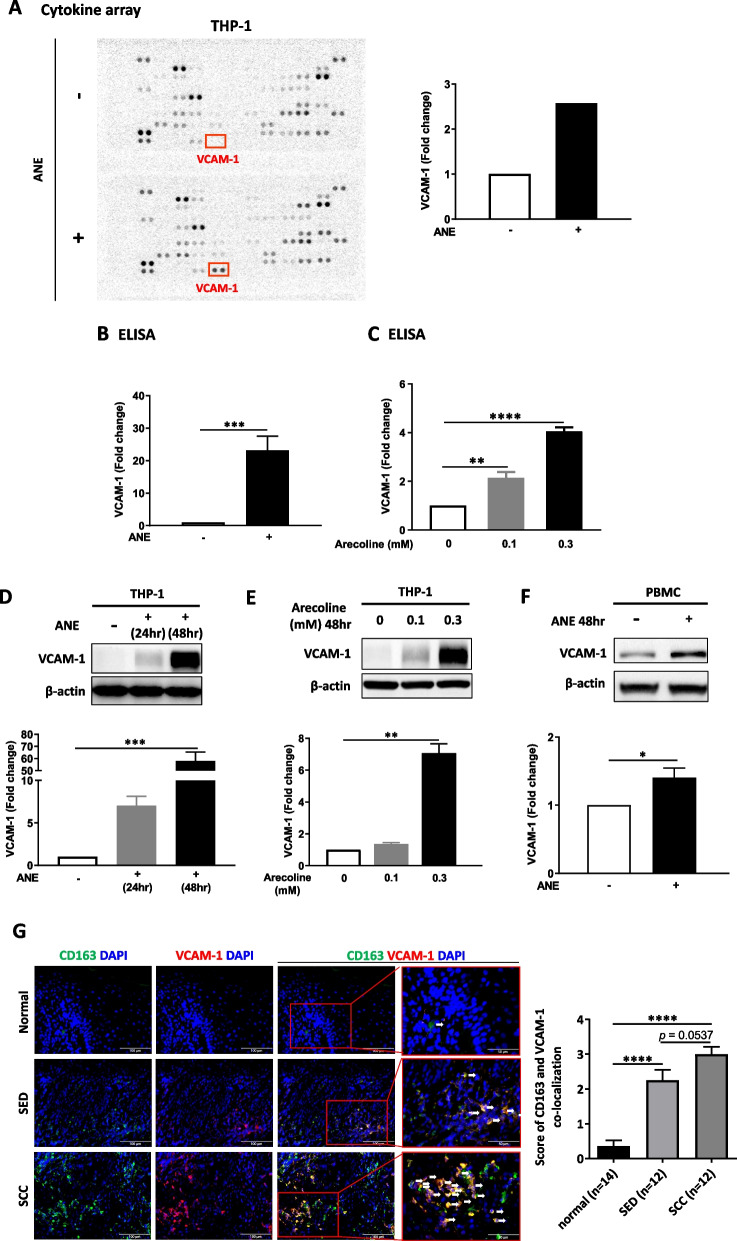
VCAM-1 was upregulated after ANE and arecoline treatment. **A** Left, Up-regulated VCAM-1 expression in ANE-treated THP-1 group, as determined by human cytokine array; Right, The quantitative figure showed VCAM-1 expression was elevated after ANE treatment. ELISA analysis of VCAM-1 expression after (**B**) ANE treatment or (**C**) arecoline treatment for 48 h. Western blot showing the expression of VCAM-1 in THP-1 cells at 24 h and 48 h after (**D**) ANE treatment or (**E**) arecoline treatment. **F** Western blot showing the expression of VCAM-1 in PBMCs after ANE treatment for 48 h. **G** Double-fluorescent immunohistochemistry for CD163 and VCAM-1 expression in oral tissue sections from patients. The merged fluorescent signal (yellow) represents colocalized CD163 (green) and VCAM-1 (red). Scale bar = 100 μm. The data are presented as the mean ± SD from three independent experiments. *, *p* < 0.05; **, *p* < 0.01; ***, *p* < 0.001; ****, *p* < 0.0001

To investigate the potential bioactive factors secreted by THP-1 cells after ANE treatment, an antibody-based proteome array was applied to screen the components in the conditioned medium of THP-1 cells. Vascular cell adhesion molecule 1 (VCAM-1) showed the greatest increase among 102 secretory proteins in the conditioned medium of ANE-treated THP-1 cells (Fig. 2A). This finding was further confirmed by the Human VCAM-1/CD106 DuoSet ELISA (Fig. 2B). In addition, the expression of VCAM-1 in the conditioned medium of arecoline-treated THP-1 cells was significantly higher than that in the untreated group (Fig. 2C). Notably, western blotting showed that the expression of VCAM-1 was 60 times higher in the conditioned medium of ANE-treated THP-1 cells than in the untreated group (Fig. 2D), and a similar result was found in the arecoline treatment group (Fig. 2E). Further study using PBMCs also showed that ANE treatment increased the expression of VCAM-1 compared to the untreated group (Fig. 2F).

Moreover, ANE-treated THP-1 cells promoted THP-1 polarization to M2 macrophages as there was a significant increase in CD163^+^ and CD206^+^ cell populations, while CD163^+^ and CD206^+^ cell populations were decreased following co-treatment with ANE and VCAM-1 inhibitor (Fig. S3A). This result suggested that VCAM-1, induced by ANE, may contribute to THP-1 M2 polarization. In agreement with these findings, a previous study reported that VCAM-1 may contribute to the development of macular fibrosis by modulating macrophage polarization and migration [38]. To confirm the in vitro findings in the clinical setting, we applied immunofluorescence staining to study the colocalization of VCAM-1 and the M2 marker CD163. The results showed that the CD163 and VCAM-1 colocalization scores were significantly higher in the SED and SCC groups than in the normal group (Fig. 2G).

### CREB phosphorylation is needed for VCAM-1 expression induced by ANE in THP-1 cells

**Fig. 3:**
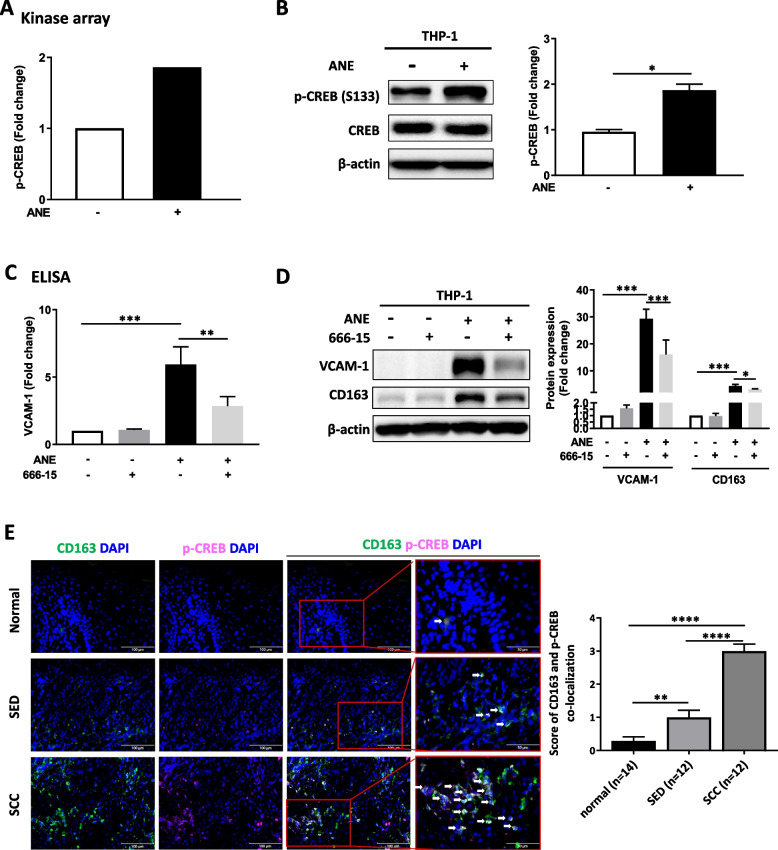
CREB is the upstream modulator of VCAM-1. **A** Phospho-kinase array analysis of THP-1 cells treated with ANE for 24 h. **B** Western blot showing the expression of p-CREB (S133) in THP-1 cells after treatment with ANE for 48 h. **C** ELISA analysis of VCAM-1 expression in THP-1 cells after ANE treatment for 48 h. **D** Western blot showing the expression of VCAM-1 and CD163 in THP-1 cells after 48 h of treatment with CREB inhibitor (666–15), ANE, or ANE combined with 30-min pretreatment with 666–15. **E** Double-fluorescent immunohistochemistry for CD163 and p-CREB expression in oral tissue sections. The merged fluorescent signal (yellow) represents colocalized CD163 (green) and p-CREB (pink). Scale bar = 100 μm. The data are presented as the mean ± SD from three independent experiments. *, *p* < 0.05; **, *p* < 0.01; ***, *p* < 0.001; ****, *p* < 0.0001

By using a phospho-kinase proteome array, we screened the potential signaling pathways activated by ANE in THP-1 cells. Increased expression of serine 133-phosphorylated CREB was observed (Figs. 3A and S3B), which was further confirmed by western blotting (Fig. 3B). Notably, ELISA result showed that ANE-induced VCAM-1 secretion in THP-1-conditioned medium was suppressed in the presence of p-CREB inhibitors, 666–15 or KG-501, as shown in Figs. 3C and S4A respectively. Western blot analysis also showed that ANE-activated VCAM-1 and CD163 expression in THP-1 cells was decreased by cotreatment with either 666–15 or KG-501 (Figs. 3D and S4B). Further immunofluorescence staining showed that the CD163 and p-CREB colocalization scores were significantly higher in the SED and SCC groups than in the normal group (Fig. 3E).

### Involvement of mitochondrial metabolism in ANE-induced VCAM-1 expression in THP-1 cells

**Fig. 4:**
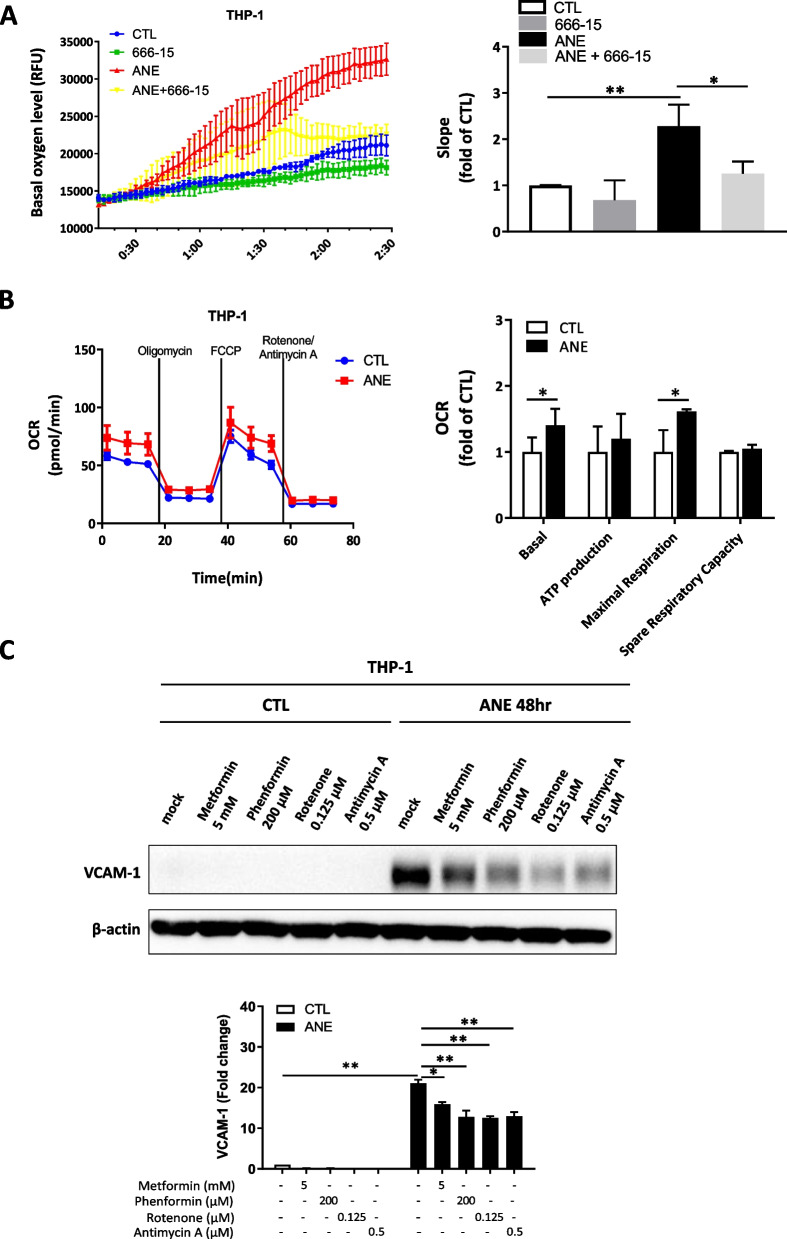
Involvement of mitochondrial metabolism in ANE-induced VCAM-1 expression in THP-1 cells. **A** The basal oxygen consumption rate in THP-1 cells was increased after 0.4 mg/ml ANE treatment for 48 h, as analyzed by the CLARIOstar Plus platform. **B** The basal oxygen consumption rate (between 0 and 15 min) in THP-1 cells was increased after ANE (0.4 mg/ml) treatment for 48 h, as analyzed by the Seahorse XF Analyzer platform. **C** The increased VCAM-1 expression in THP-1 cells treated with ANE for 48 h was suppressed in the presence of mitochondrial complex I inhibitors (metformin, phenformin, and rotenone) and a mitochondrial complex III inhibitor (antimycin A). The data are presented as the mean ± SD from three independent experiments. *, *p* < 0.05; **, *p* < 0.01

Recent studies have shown that mitochondrial metabolism plays a role in macrophage polarization, differentiation, and survival [39, 40]. Using the CLARIOstar plus platform (Fig. 4A) and Seahorse XF Analyzer (Fig. 4B), we observed that THP-1 cells had a significantly increased oxygen consumption rate (OCR) after ANE treatment. However, cotreatment with the CREB inhibitor 666–15 reduced the OCR induced by ANE treatment (Fig. 4A and B). Western blotting result showed that ANE-induced VCAM-1 expression was suppressed in the presence of antimycin A, a mitochondrial electron complex III inhibitor, and rotenone, a mitochondrial electron complex I inhibitor, while there was only a modest decline in VCAM-1 expression in the presence of metformin and phenformin (Fig. 4C). Metformin acts as a weak yet targeted inhibitor of complex I, while phenformin exerts a more potent but less selective influence on the mitochondrial electron transport chain [41]. Notably, metformin's activity is relatively weak compared to rotenone [42, 43]. Taken together, our study suggests that rotenone or antimycin A may be more potent inhibitors of VCAM-1 expression.

### Conditioned medium from ANE-treated THP-1 cells and PBMCs promoted migration, invasion, and mesenchymal phenotypes in oral precancer cells

**Fig. 5:**
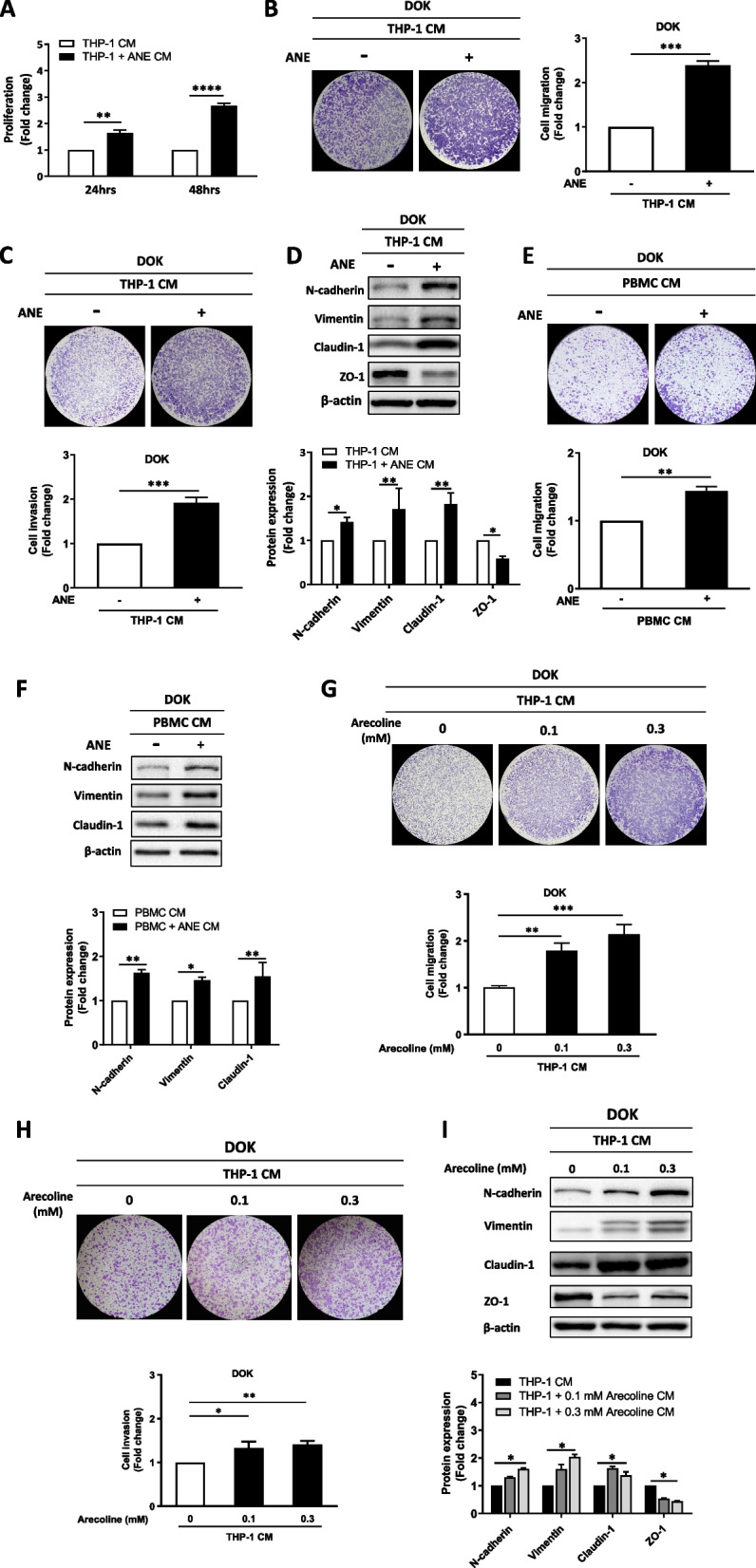
Conditioned medium from ANE-treated THP-1 monocytes promoted migration ability and mesenchymal phenotypes in DOK oral precancer cells. **A** Proliferation activity of DOK cells after treatment with THP-1-conditioned medium for 24 h and 48 h, as determined by the XTT assay. DOK cells were treated with THP-1 conditioned medium for 48 h, **B** cell migration and (**C**) cell invasion were analyzed by transwell assay after 72 h. **D** Expression of EMT markers in DOK cells after treatment with THP-1 conditioned medium for 48 h. **E** DOK cells were treated with PBMC-conditioned medium for 48 h, and cell migration was analyzed by transwell migration assay after 72 h. **F** Expression of EMT markers in DOK cells after treatment with PBMC-conditioned medium for 48 h. DOK cells were treated with THP-1 conditioned medium for 48 h, **G** cell migration and (H) cell invasion were analyzed by transwell assay after 72 h. **I** Expression of EMT markers in DOK cells after treatment with THP-1 conditioned medium for 48 h. The data are presented as the mean ± SD from three independent experiments. *, *p* < 0.05; **, *p* < 0.01; ***, *p* < 0.001; ****, *p* < 0.0001

To investigate the biological effects of ANE-treated THP-1 cells on DOK oral precancer cells, conditioned medium (CM) from THP-1 cells was used to treat DOK cells, followed by XTT cell proliferation assays, transwell migration assays and western blot analysis for epithelial-mesenchymal transition (EMT) markers. Treatment of DOK cells with CM from ANE-treated THP-1 cells increased DOK proliferation (Fig. 5A), migration and invasion (Fig. 5B, C) and expression of mesenchymal markers, including N-cadherin, vimentin, and claudin-1, in DOK cells (Fig. 5D). In contrast, the expression of the epithelial marker ZO-1 was decreased (Fig. 5D). These results were further confirmed by using PBMCs, and increased migration ability was observed in DOK cells after treatment with CM from ANE-treated PBMCs (Fig. 5E). Additionally, the expression of N-cadherin, vimentin, and claudin-1 was increased in DOK cells after treatment with CM from ANE-treated PBMCs (Fig. 5F). Similarly, increased cell migration and cell invasion (Fig. 5G, H), increased expression of mesenchymal markers and decreased expression of epithelial markers (Fig. 5I) were observed in DOK cells after treatment with CM from arecoline treated THP-1 cells.

### VCAM-1 secreted from THP-1 cells is a major factor in regulating oral precancer cell migration

**Fig. 6:**
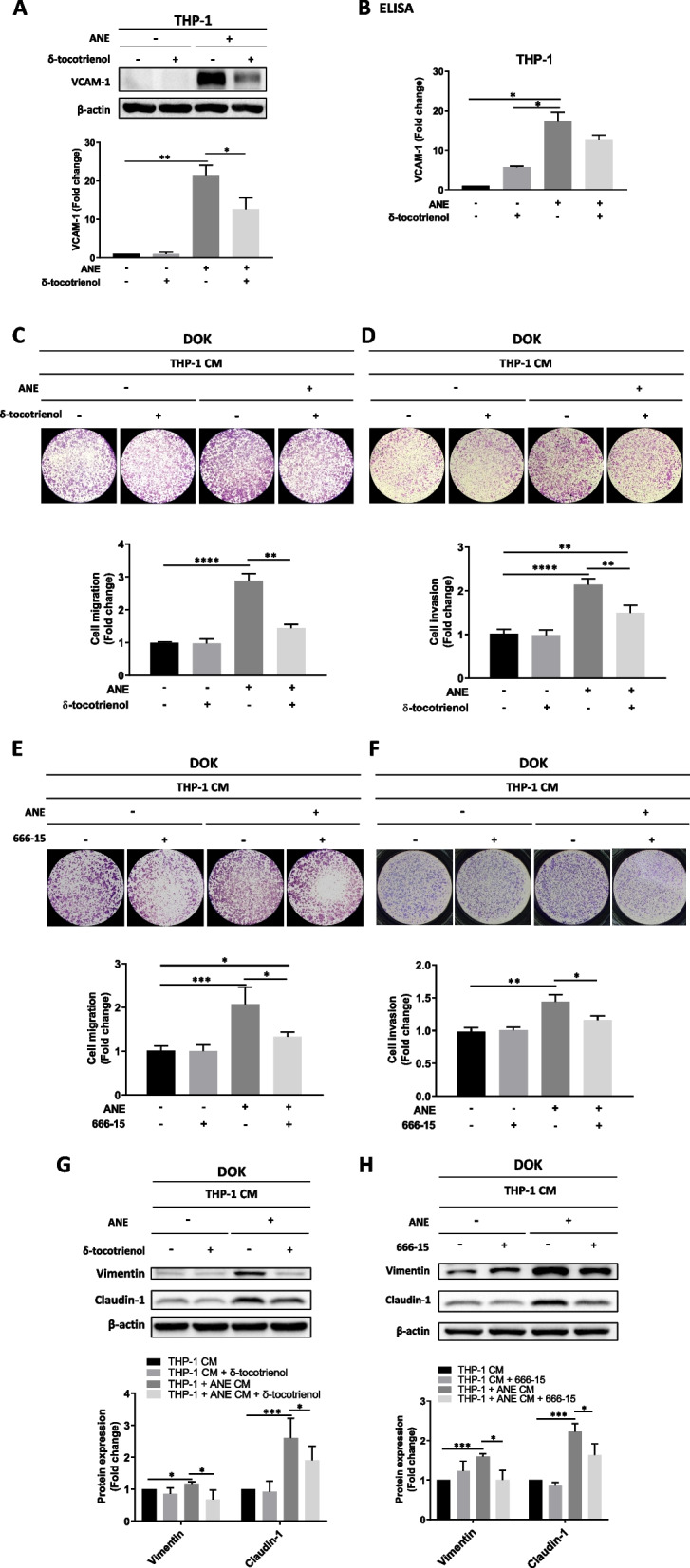
VCAM-1 secreted by THP-1 cells after ANE treatment was involved in promoting DOK cell migration. **A** The expression of VCAM-1 in THP-1 cells after 48 h of treatment with a VCAM-1 inhibitor (δ-tocotrienol), ANE, or ANE combined with 30 min of pretreatment with δ-tocotrienol. **B** ELISA analysis of VCAM-1 production by THP-1 cells after 48 h of treatment with a VCAM-1 inhibitor (δ-tocotrienol), ANE, or ANE combined with 30 min of pretreatment with δ-tocotrienol. DOK cells were treated with the indicated conditioned medium for 48 h and then seeded on transwell plates for 72 h for (**C**) cell migration analysis and (**D**) cell invasion analysis. DOK cells were treated with the indicated conditioned medium for 48 h and then seeded on transwell plates for 72 h for (**E**) cell migration analysis and (**F**) cell invasion analysis. Expression of mesenchymal markers in DOK cells after treatment with the indicated conditioned media for 48 h in the presence or absence of (**G**) VCAM-1 inhibitor (δ-tocotrienol) or (**H**) p-CREB inhibitor (666–15). The data are presented as the mean ± SD from three independent experiments. *, *p* < 0.05; **, *p* < 0.01; ***, *p* < 0.001; ****, *p* < 0.0001

We further examined whether blockage of the ANE-induced p-CREB/VCAM-1 pathway in THP-1 cells affects the malignant behaviors of DOK cells. Treatment of THP-1 cells with δ-tocotrienol, a well-known VCAM-1 inhibitor [44], led to decreased expression of VCAM-1 in ANE-treated THP-1 cells, as determined by western blot (Fig. 6A). Similar results were observed by ELISA (Fig. 6B). Further study showed that the increased DOK migration and invasion induced by CM from ANE-treated THP-1 cells was diminished by cotreatment with either δ-tocotrienol (Fig. 6C, D) or 666–15 (Fig. 6E, F). Additionally, western blot analysis showed that cotreatment of DOK cells with either δ-tocotrienol or 666–15 significantly diminished the increased expression of vimentin and claudin-1 induced by CM from ANE-treated THP-1 cells (Fig. 6G, H).

### ANE enhanced M2 polarization and related signaling pathways in the oral buccal tissue of hamsters

**Fig. 7:**
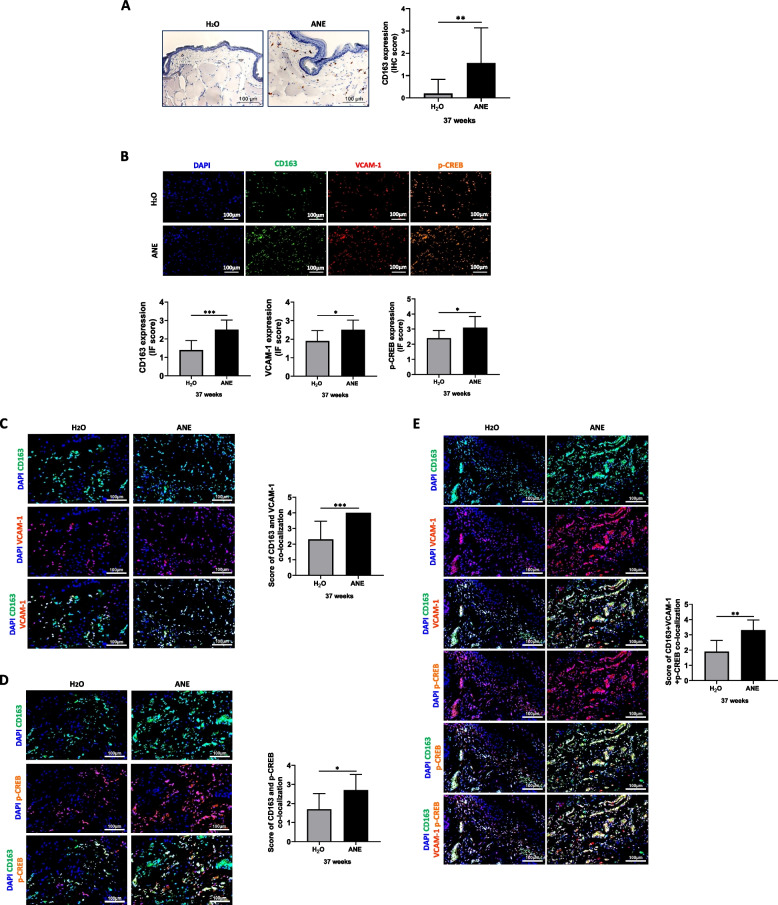
Effects of ANE on CD163 expression and related signaling pathways in the hamster buccal pouch model. **A** Representative photos showing that the expression of CD163 was significantly increased after ANE treatment, as determined by immunohistochemical staining. Scale bar = 100 μm. **B** Fluorescence immunohistochemistry and scores for CD163, VCAM-1 and p-CREB expression in the buccal pouch of hamsters. **D** Double-fluorescence immunohistochemistry for coexpression of CD163 and VCAM-1 and coexpression of CD163 and p-CREB in the buccal pouch of hamsters. **E** Triple-staining immunohistochemistry for CD163, VCAM-1 and p-CREB in the buccal pouch of hamsters. The merged fluorescent signal (yellow) represents colocalized CD163 (green), VCAM-1 (red), and p-CREB (orange). Scale bar = 100 μm. The data are presented as the mean ± SD from three independent experiments. *, *p* < 0.05; **, *p* < 0.01; ***, *p* < 0.001

Finally, we established an in vivo model using the buccal pouch of hamsters painted with ANE. After 37 weeks of treatment, we observed that the population of CD163^+^ cells was significantly increased in the ANE-treated group compared to the untreated group (Fig. 7A). In agreement with the in vitro data, triple-fluorescence immunohistochemistry confirmed that tissue specimens from the ANE-treated group had a higher colocalization of CD163/VCAM-1/p-CREB than those from the untreated group (Fig. 7B-E).

## Discussion

**Fig. 8:**
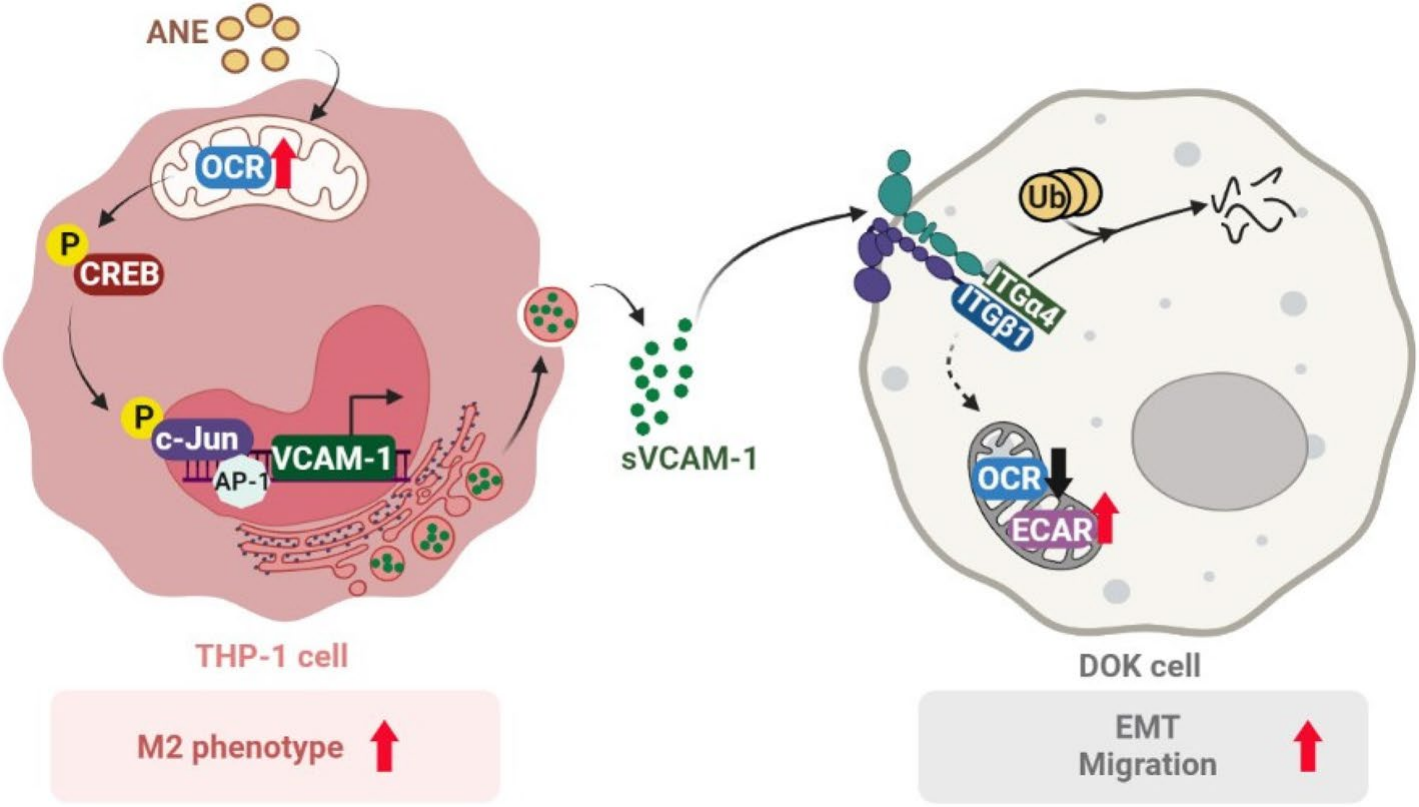
Schematic drawing for malignant transformation in oral premalignant cells via ANE-induced M2 macrophage differentiation

This is the first study to explore the effects of areca nut extract and its secreted factors in modulating the immune system during oral carcinogenesis. Areca nut extract upregulates mitochondrial metabolism and p-CREB/VCAM-1 signaling in macrophages, followed by ubiquitination of integrin α4/β1 (ITGα4/ITGβ1), decreased mitochondrial metabolism and increased EMT and migration in oral precancer cells. Together, these findings depict a malignant transformation model mediated by ANE-induced oral tumor microenvironmental modulation (Fig. 8).

There are several reported risk factors for oral cancer. Although human papillomavirus (HPV) causes cancers in the mouth and throat [45, 46], leading to 70% of oral cancer cases in the United States, areca nut is the main risk factor in Taiwan [22]. HPV and areca nut extract are independent prognostic factors for disease-free and overall survival in oropharyngeal cancer patients [47, 48]. In Taiwan, the incidence of betel nut-associated head and neck cancer is still growing, and only one-third of OSCC cases are HPV-positive [49]. The main ingredients in areca nut include arecoline, arecaidine, guvacine, and guvacoline (Fig. S2B).

### Arecoline promotes precancer cell migration and M2 polarization

Arecoline promotes oral cancer through genotoxic, mutagenic, and carcinogenic effects. Arecoline increases malignant esophageal tumors in mice and benign esophageal and tongue tumors in rats [50]. Interestingly, our data showed that the arecoline concentration in conditioned medium was significantly reduced when cultured with THP-1 cells for 48 h (Fig. S2D), and the conditioned medium promoted precancer cell migration ability (Fig. 5B). Furthermore, we observed increased M2 macrophage polarization in the hamster buccal pouch after 37 weeks of treatment with areca nut extract (Fig. 7A). Together, these findings suggest the involvement of M2 macrophages in areca nut-mediated oral carcinogenesis.

### Upregulation of mitochondrial respiration with ANE treatment

Mitochondrial respiration is an essential indicator of cellular function [51, 52], with HPV status influencing metabolic profiles in oral cancer. HPV-negative cases are associated with activation in all energy pathways, with upregulated expression of genes associated with glycolysis and oxidative phosphorylation [53]. In the current study, we showed that ANE treatment significantly increased the oxygen consumption rate of THP-1 cells, including increases in basal respiration, ATP production, maximal respiration, and spare respiratory capacity (Fig. 4B). This upregulated activation in mitochondrial metabolism and energetic reserves in THP-1 cells may indirectly contribute to enhanced DOK cell migration (Fig. 5B and D).

### Conditioned medium from ANE-treated THP-1 cells promotes oral precancer cell EMT via VCAM-1

We explored the key molecules regulating macrophage differentiation after ANE treatment. Previously, CREB was found to enhance macrophage survival and M2 differentiation via NF-κB [54]. In this study, we found that inactivation of p-CREB suppressed the OCR induced by ANE treatment (Fig. 4A). In addition, inhibition of p-CREB resulted in decreased expression of p–c-Jun (Fig. S5A), a key molecule in activating the transcription factor AP-1, which may lead to decreased VCAM-1 expression [55]. AP-1 is a crucial transcription factor that regulates the expression of downstream genes which involved in various aspects of cancer biology, including cell growth, migration, invasion, metastasis and drug resistance [56]. During arthritis progression, AP-1 controls VCAM-1 production and cell motility [57]. In addition, suppression of c-jun phosphorylation resulted in reduced AP-1 and VCAM-1 expression in osteoarthritis synovial fibroblasts [58]. In the current study, we found that p–c-Jun expression was decreased in the presence of p-CREB inhibitor.

VCAM-1, a member of the immunoglobulin superfamily, is closely related to the development of malignant tumors, such as breast [59], ovarian [60], and clear cell renal carcinoma [61]. Overexpression of VCAM-1 results in lung or bone metastasis via recruitment of monocytes and macrophages and formation of a complex that promotes evasion of circulating tumor cells from immune attack and facilitates transendothelial migration [62]. Interestingly, we observed that ANE treatment activated the p-CREB/VCAM-1 pathway and M2 polarization in THP-1 cells. Using Human VCAM-1/CD106 DuoSet ELISA, we observed increased expression of VCAM-1 in the conditioned medium of ANE-treated THP-1 cells (Fig. 2B). We also found that ANE induced upregulation of EMT, which promotes tumor plasticity by transforming epithelial cells into mesenchymal cells via the functional loss of cell adhesion and the attainment of migratory and invasive properties [63, 64]. Furthermore, conditioned medium from ANE-treated THP-1 cells increased migration ability (Fig. 5B) and the protein expression of N-cadherin, vimentin, and claudin-1 in DOK cells (Fig. 5C). Notably, these effects were suppressed by the CREB inhibitor 666–15 and the VCAM-1 inhibitor δ-tocotrienol (Fig. 6A, B, E, F). Collectively, our results suggest that secreted VCAM-1 is a key factor in the conditioned medium of ANE-treated THP-1 cells and promotes oral precancer cell migration through an EMT-associated mechanism.

Data analysis from TCGA and Gene Expression Omnibus databases showed that high VCAM-1 expression was correlated with metastasis and short survival in colorectal cancer patients [65]. VCAM-1 has also been reported as an influential factor in the invasion and metastasis of colorectal cancer cells by facilitating the formation of pseudopodia and invadopodia to cross the vessel wall [65]. VCAM-1 is a ligand for α4β1 integrin (VLA-4), and sVCAM-1 binding to α4 integrin is cell-type-specific and energy dependent [66, 67]. The interaction of sVCAM-1 with integrin α4β1 on tumor cells plays a role in tumor cell extravasation [66]. In this study, secreted sVCAM-1 from ANE-treated THP-1 cells induced integrin α4 ubiquitination (Fig. S5B) and decreased the oxygen consumption rate but increased the extracellular acidification rate (Fig. S6) in DOK cells. Further studies are needed to explore the detailed mechanisms by which sVCAM-1 interacts with α4β1 integrin in the regulation of mitochondrial metabolism.

The in vitro evidence that ANE induces M2 macrophage differentiation to promote malignant behaviors of oral premalignant cells was further supported by the in vivo study, which found that the ANE-treated group showed higher CD163 M2 marker expression than the untreated group (Fig. 7A). Further immunohistochemistry analysis also demonstrated that oral buccal tissues in the ANE-treated group had a higher level of colocalized CD163/VCAM-1/p-CREB than those in the untreated group (Fig. 7B-D).

### Novel Therapeutic Targets in betel quid chewing

Prevention of the transformation of oral potentially malignant disorders (OPMD) to oral squamous cell carcinoma (OSCC) is critical in reducing the burden of disease related to betel quid chewing, particularly in South and Southeast Asia [14, 15]. In this regard, the data presented here elucidate some of the pathways by which ANE affects these changes in OPMD and provide novel therapeutic targets. These include the recent development of novel prodrugs of the CREB inhibitor 666–15, with increased oral bioavailability and efficacy in preclinical breast cancer models [68].

### Bias and limitations

However, this study has a limitation due to the lack of a significant difference in pathologic progression between the ANE-treated group and the untreated group in the hamster model (data not shown). In agreement with our study, a previous study observed focal epithelial dysplasia only in 10% of hamster buccal pouches after areca nut chewing for 18 months [69]*.* To solve this problem, the treatment time may need to be prolonged or co-treated with DMBA alternatively [70, 71]. In this current study, 0.5% DMBA was also used to initiate malignant transformation, our data demonstrated that co-treatment DMBA and ANE group tends to have more severe pathologic changes than DMBA alone group (Fig. S7A and B). Also, the expression of CD163 and p-CREB was higher in hamster group painted with both DMBA and ANE at week-8 and week-10 compared to that of control group or DMBA alone group (Fig. S7C and D). However, a lower DMBA concentration may be required to stand out the pathologic effects of ANE [72]. Moreover, we found that the p-CREB/VCAM-1 pathway was activated and THP-1 cells were polarized to M2 macrophages in the presence of ANE. Thus, an experimental design using animal model with blockage of either M2 polarization or VCAM-1 may be necessary to explore the effects of ANE on malignant transformation.

## Conclusion

The present study provides in vitro, in vivo and clinical evidence that areca nut—and particularly its active constituent arecoline—act via induction of M2 macrophage differentiation and secretion of oncogenic cytokines in the oral tumor microenvironment, promoting malignant transformation of oral premalignant cells.

### Supplementary Information

Additional file 1. Supplementary figures.

Additional file 2

## Data Availability

The datasets used and/or analyzed during the current study are available from the corresponding author upon reasonable request.

## Abbreviations

ANE: Areca nut extract
ANOVA: One-way analysis of variance
ATP: Adenosine triphosphate
CM: Conditioned medium
Co-IP: Co-immunoprecipitation
CREB: CAMP-response element binding
CXCL1: Chemokine (C-X-C motif) ligand 1
ddH_2_O: Double distilled water
DMBA: 7,12-Dimethylbenz[a]anthracene
DMEM: Dulbecco’s Modified Eagle Medium
ECAR: Extracellular acidification rate
EGFR: Epidermal growth factor receptor
ELISA: Enzyme-linked immunosorbent assay
EMT: Epithelial-mesenchymal transition
ETC: Electron transport chain
FCCP: Carbonyl cyanide-4 (trifluoromethoxy) phenylhydrazone
H&E: Hematoxylin and Eosin
HPV: Human papillomavirus
IARC: International Agency for Research on Cancer
IF: Immunofluorescence
IHC: Immunohistochemistry
IL-1RA: Interleukin-1 receptor antagonist
IRB: Institutional Review Board
ITGα4: Integrin α4
ITGβ1: Integrin β1
MED: Mild epithelial dysplasia
MoED: Moderate epithelial dysplasia
OCR: Oxygen consumption rate
OD: Optical density
OPMD: Oral potentially malignant disorders
OSCC: Oral squamous cell carcinoma
PBMCs: Peripheral blood mononuclear cells
PBS: Phosphate-buffered saline
PI: Propidium iodide
PMA: Methoxy propyl acetate
RPMI: Roswell Park Memorial Institute
SED: Severe epithelial dysplasia
SPSS: Statistical Package for the Social Science
sVCAM-1: Soluble vascular cell adhesion molecule-1
TAMs: Tumor-associated macrophages
TCGA: The Cancer Genome Atlas
VCAM-1: Vascular cell adhesion molecule 1
VLA-4: Integrin α4β1 (very late antigen-4)
XTT: 2,3-Bis-(2-methoxy-4-nitro-5-sulfophenyl)-2H-tetrazolium-5-carboxanilide)
ZO-1: Zona occludens 1
