# Supplementary material for: Areca nut-induced metabolic reprogramming and M2 differentiation promote OPMD malignant transformation
Source: J Exp Clin Cancer Res. 2024 Aug 20;43:233. doi: 10.1186/s13046-024-03163-z (PMC11334407; doi:10.1186/s13046-024-03163-z)
Supplement: Supplementary file 1 — Additional file 1. Supplementary figures. [file 13046_2024_3163_MOESM1_ESM.pptx]

## Slide 1
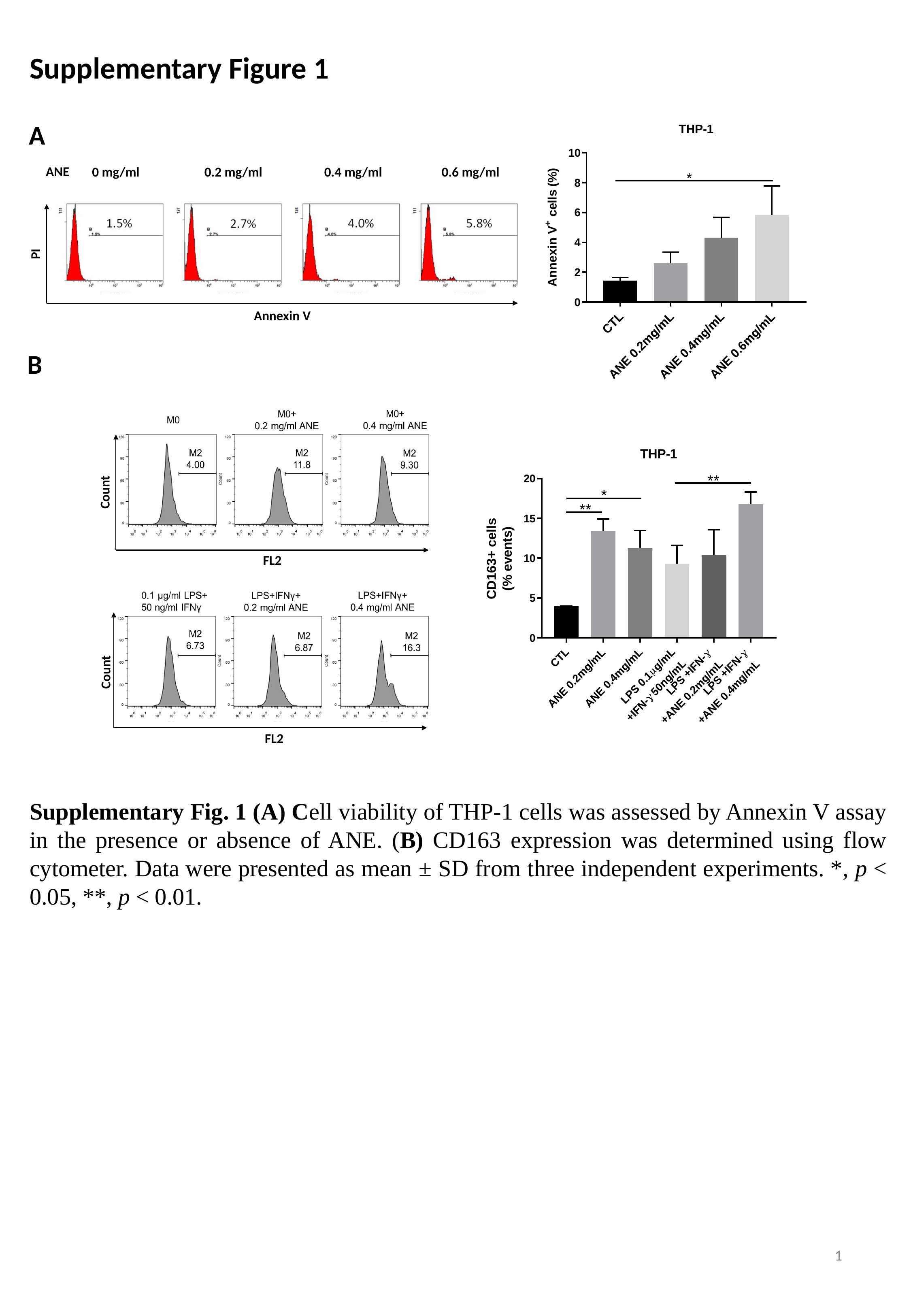

Supplementary Figure 1
A
ANE
0 mg/ml
0.2 mg/ml
0.4 mg/ml
0.6 mg/ml
PI
Annexin V
B
Count
FL2
Count
FL2
Supplementary Fig. 1 (A) Cell viability of THP-1 cells was assessed by Annexin V assay in the presence or absence of ANE. (B) CD163 expression was determined using flow cytometer. Data were presented as mean ± SD from three independent experiments. *, p < 0.05, **, p < 0.01.
1

## Slide 2
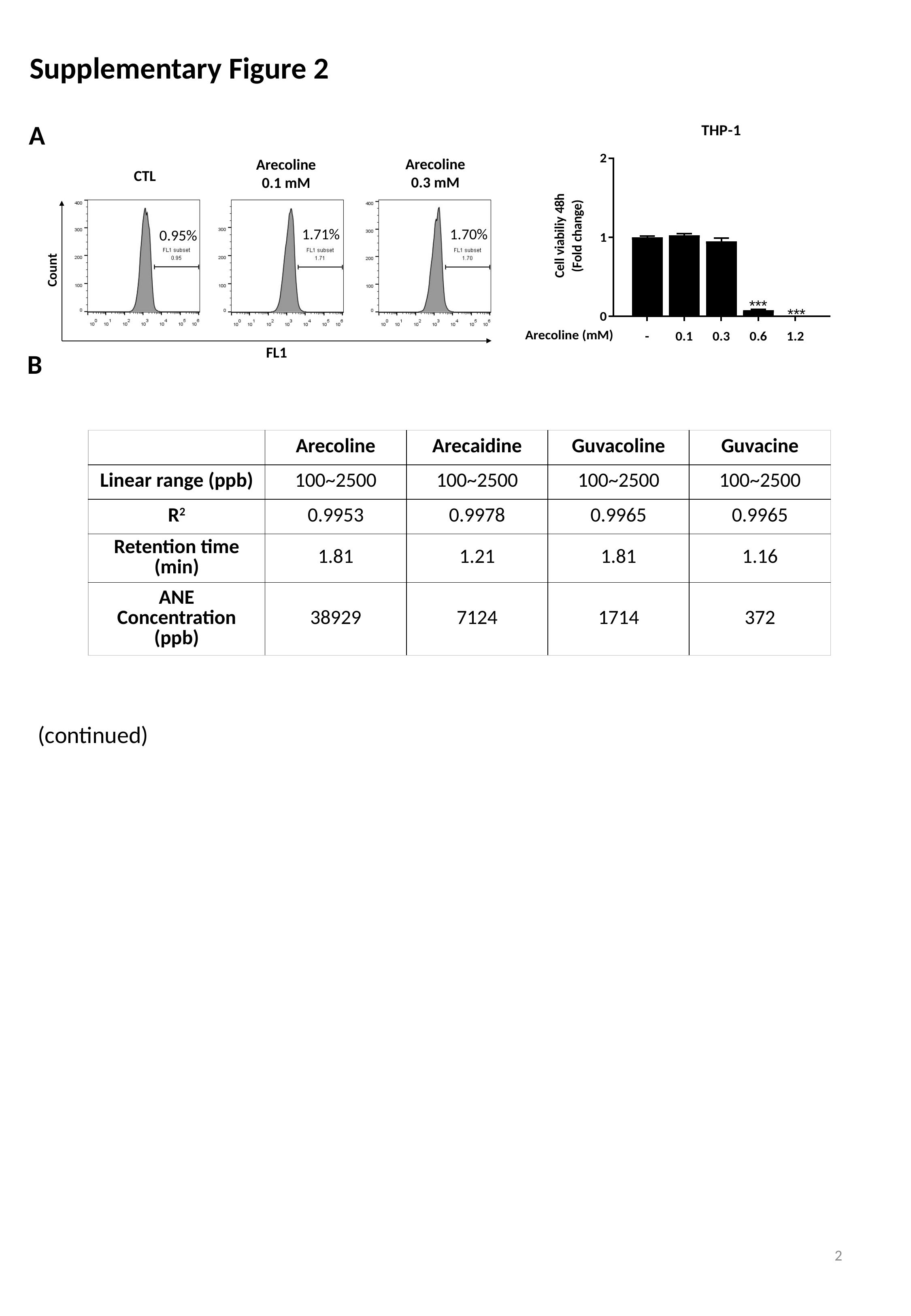

Supplementary Figure 2
A
Arecoline
0.3 mM
Arecoline
0.1 mM
CTL
1.71%
1.70%
0.95%
Count
FL1
B
| | Arecoline | Arecaidine | Guvacoline | Guvacine |
| --- | --- | --- | --- | --- |
| Linear range (ppb) | 100~2500 | 100~2500 | 100~2500 | 100~2500 |
| R2 | 0.9953 | 0.9978 | 0.9965 | 0.9965 |
| Retention time (min) | 1.81 | 1.21 | 1.81 | 1.16 |
| ANE Concentration (ppb) | 38929 | 7124 | 1714 | 372 |
(continued)
2

## Slide 3
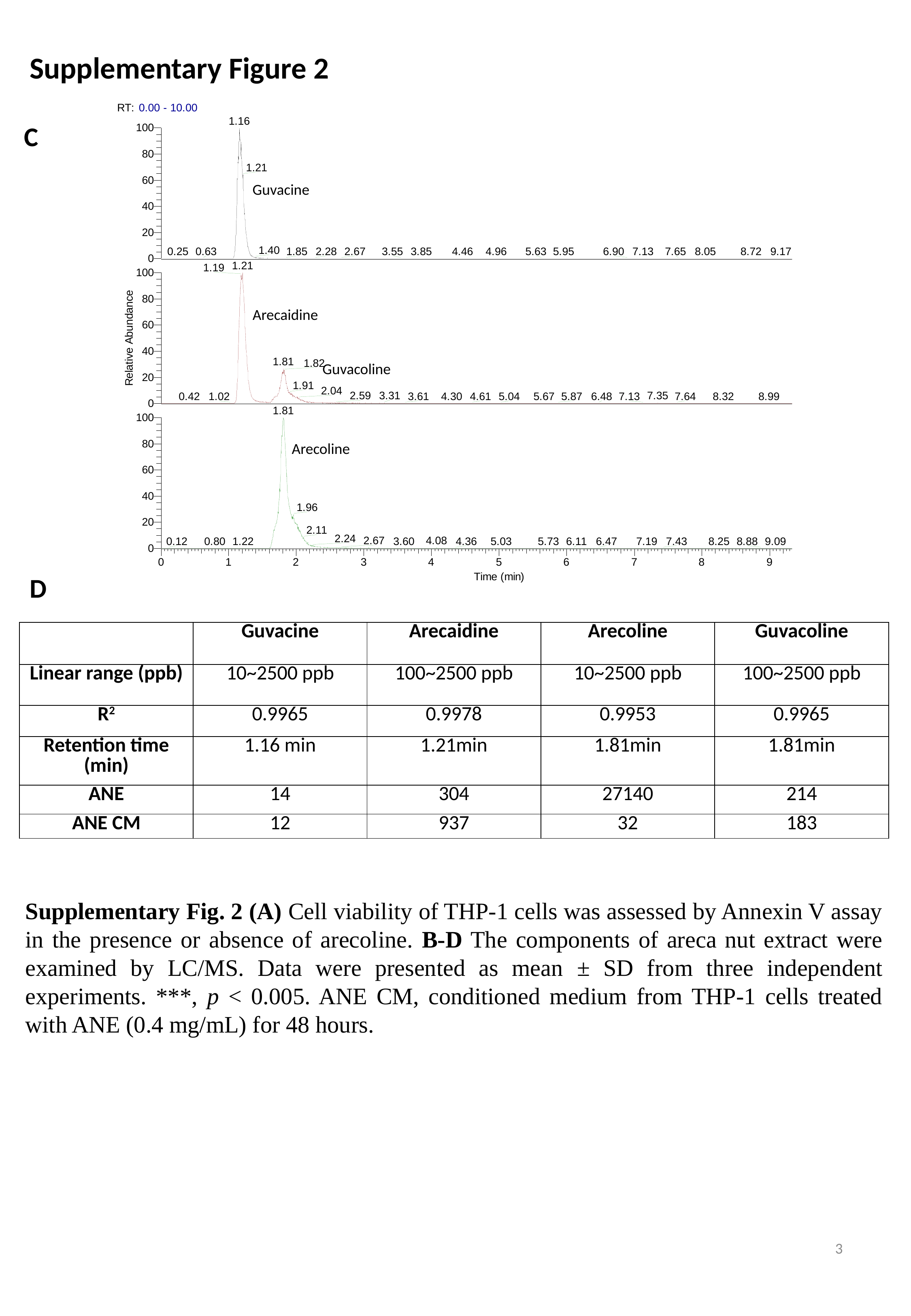

Supplementary Figure 2
Guvacine
Arecaidine
Guvacoline
Arecoline
C
D
| | Guvacine | Arecaidine | Arecoline | Guvacoline |
| --- | --- | --- | --- | --- |
| Linear range (ppb) | 10~2500 ppb | 100~2500 ppb | 10~2500 ppb | 100~2500 ppb |
| R2 | 0.9965 | 0.9978 | 0.9953 | 0.9965 |
| Retention time (min) | 1.16 min | 1.21min | 1.81min | 1.81min |
| ANE | 14 | 304 | 27140 | 214 |
| ANE CM | 12 | 937 | 32 | 183 |
Supplementary Fig. 2 (A) Cell viability of THP-1 cells was assessed by Annexin V assay in the presence or absence of arecoline. B-D The components of areca nut extract were examined by LC/MS. Data were presented as mean ± SD from three independent experiments. ***, p < 0.005. ANE CM, conditioned medium from THP-1 cells treated with ANE (0.4 mg/mL) for 48 hours.
3

## Slide 4
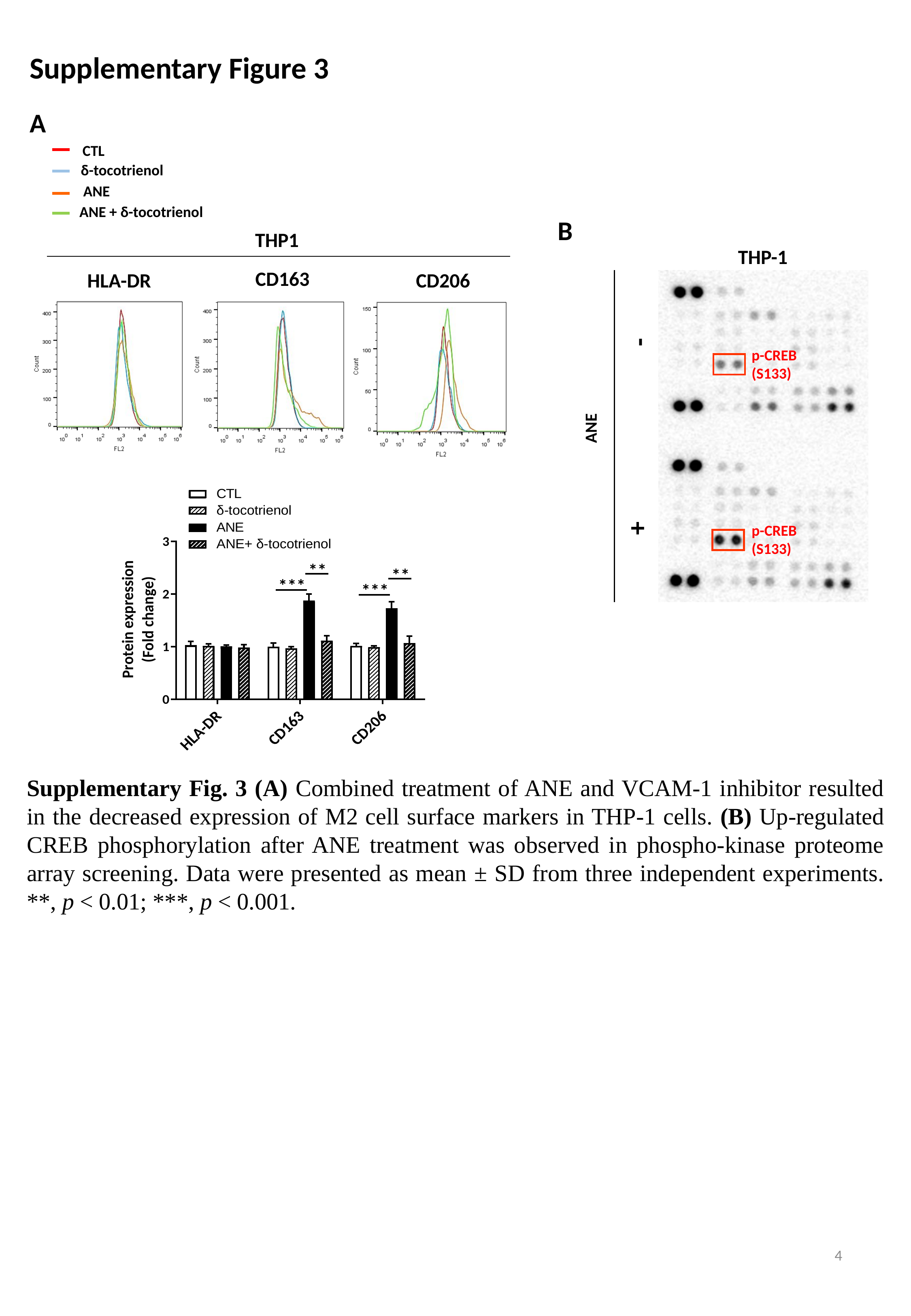

Supplementary Figure 3
A
CTL
δ-tocotrienol
ANE
ANE + δ-tocotrienol
B
THP1
THP-1
-
p-CREB
(S133)
ANE
+
p-CREB
(S133)
CD163
HLA-DR
CD206
Supplementary Fig. 3 (A) Combined treatment of ANE and VCAM-1 inhibitor resulted in the decreased expression of M2 cell surface markers in THP-1 cells. (B) Up-regulated CREB phosphorylation after ANE treatment was observed in phospho-kinase proteome array screening. Data were presented as mean ± SD from three independent experiments. **, p < 0.01; ***, p < 0.001.
4

## Slide 5
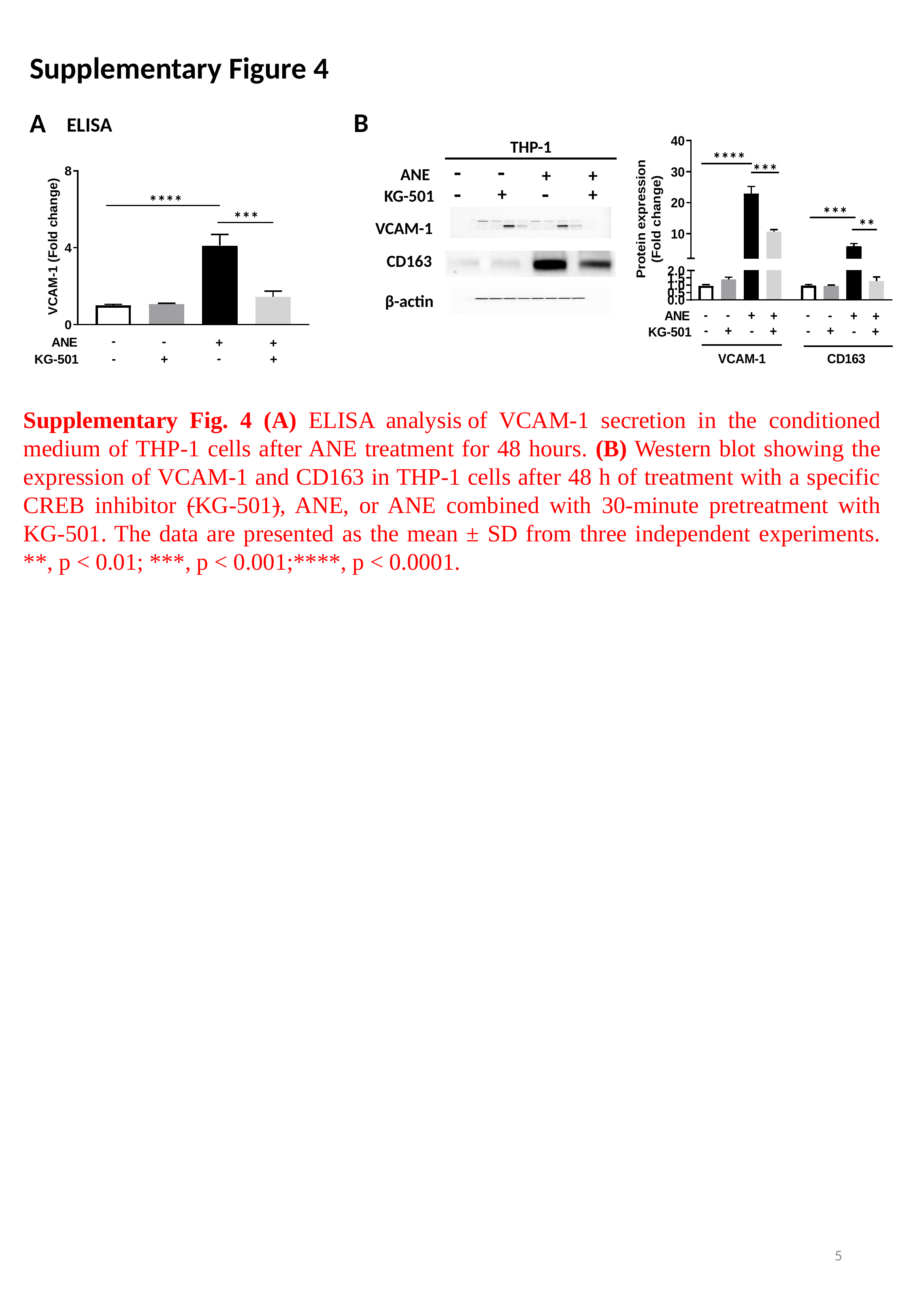

Supplementary Figure 4
B
A
ELISA
THP-1
-
-
+
+
ANE
-
-
+
+
KG-501
VCAM-1
CD163
β-actin
Supplementary Fig. 4 (A) ELISA analysis of VCAM-1 secretion in the conditioned medium of THP-1 cells after ANE treatment for 48 hours. (B) Western blot showing the expression of VCAM-1 and CD163 in THP-1 cells after 48 h of treatment with a specific CREB inhibitor (KG-501), ANE, or ANE combined with 30-minute pretreatment with KG-501. The data are presented as the mean ± SD from three independent experiments. **, p < 0.01; ***, p < 0.001;****, p < 0.0001.
5

## Slide 6
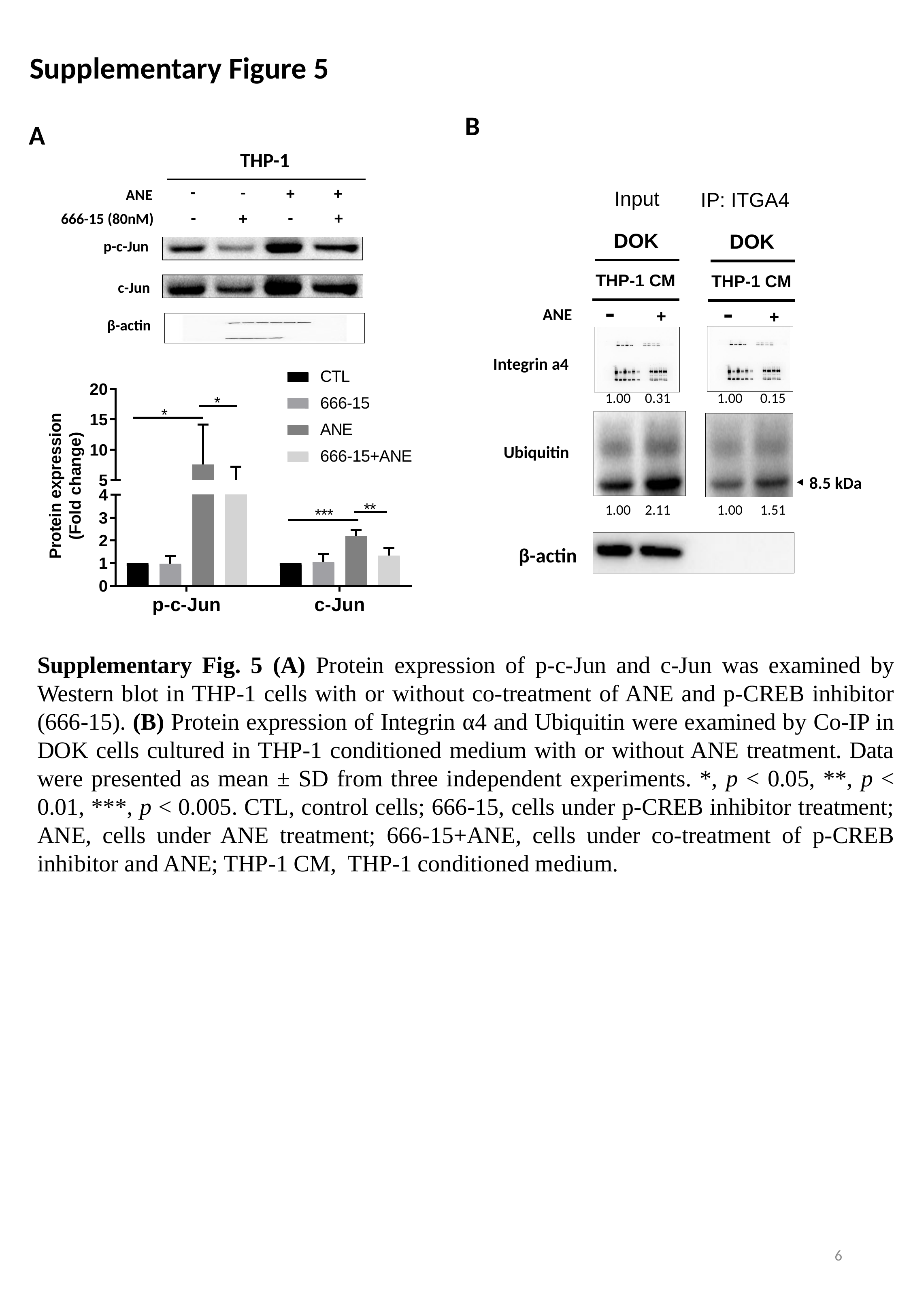

Supplementary Figure 5
B
A
THP-1
-
-
+
+
ANE
Input
IP: ITGA4
DOK
DOK
THP-1 CM
THP-1 CM
-
-
ANE
+
+
Integrin a4
Ubiquitin
β-actin
-
+
-
+
666-15 (80nM)
p-c-Jun
c-Jun
β-actin
| 1.00 | 0.31 | | 1.00 | 0.15 |
| --- | --- | --- | --- | --- |
8.5 kDa
| 1.00 | 2.11 | | 1.00 | 1.51 |
| --- | --- | --- | --- | --- |
Supplementary Fig. 5 (A) Protein expression of p-c-Jun and c-Jun was examined by Western blot in THP-1 cells with or without co-treatment of ANE and p-CREB inhibitor (666-15). (B) Protein expression of Integrin α4 and Ubiquitin were examined by Co-IP in DOK cells cultured in THP-1 conditioned medium with or without ANE treatment. Data were presented as mean ± SD from three independent experiments. *, p < 0.05, **, p < 0.01, ***, p < 0.005. CTL, control cells; 666-15, cells under p-CREB inhibitor treatment; ANE, cells under ANE treatment; 666-15+ANE, cells under co-treatment of p-CREB inhibitor and ANE; THP-1 CM, THP-1 conditioned medium.
6

## Slide 7
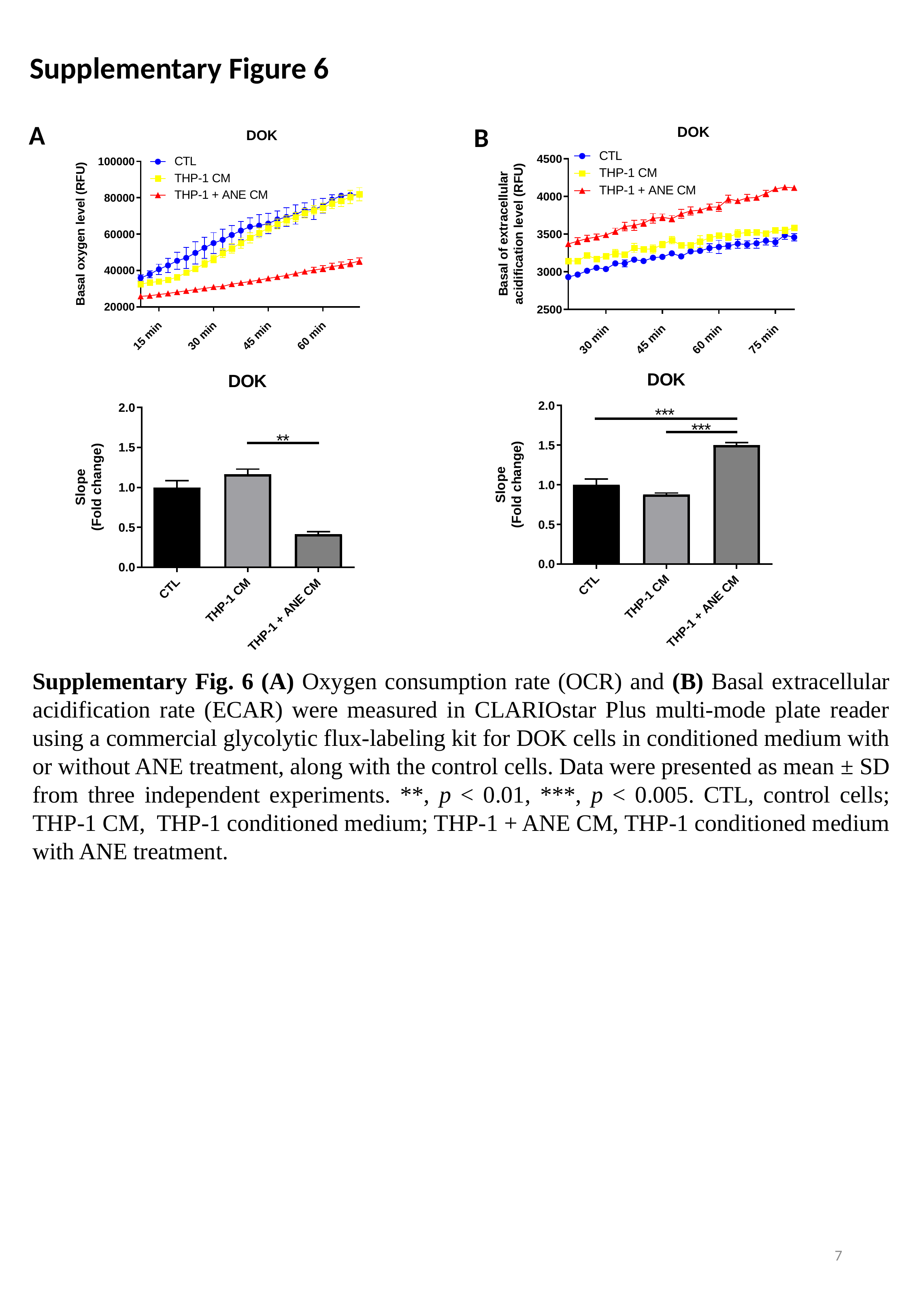

Supplementary Figure 6
A
B
Supplementary Fig. 6 (A) Oxygen consumption rate (OCR) and (B) Basal extracellular acidification rate (ECAR) were measured in CLARIOstar Plus multi-mode plate reader using a commercial glycolytic flux-labeling kit for DOK cells in conditioned medium with or without ANE treatment, along with the control cells. Data were presented as mean ± SD from three independent experiments. **, p < 0.01, ***, p < 0.005. CTL, control cells; THP-1 CM, THP-1 conditioned medium; THP-1 + ANE CM, THP-1 conditioned medium with ANE treatment.
7

## Slide 8
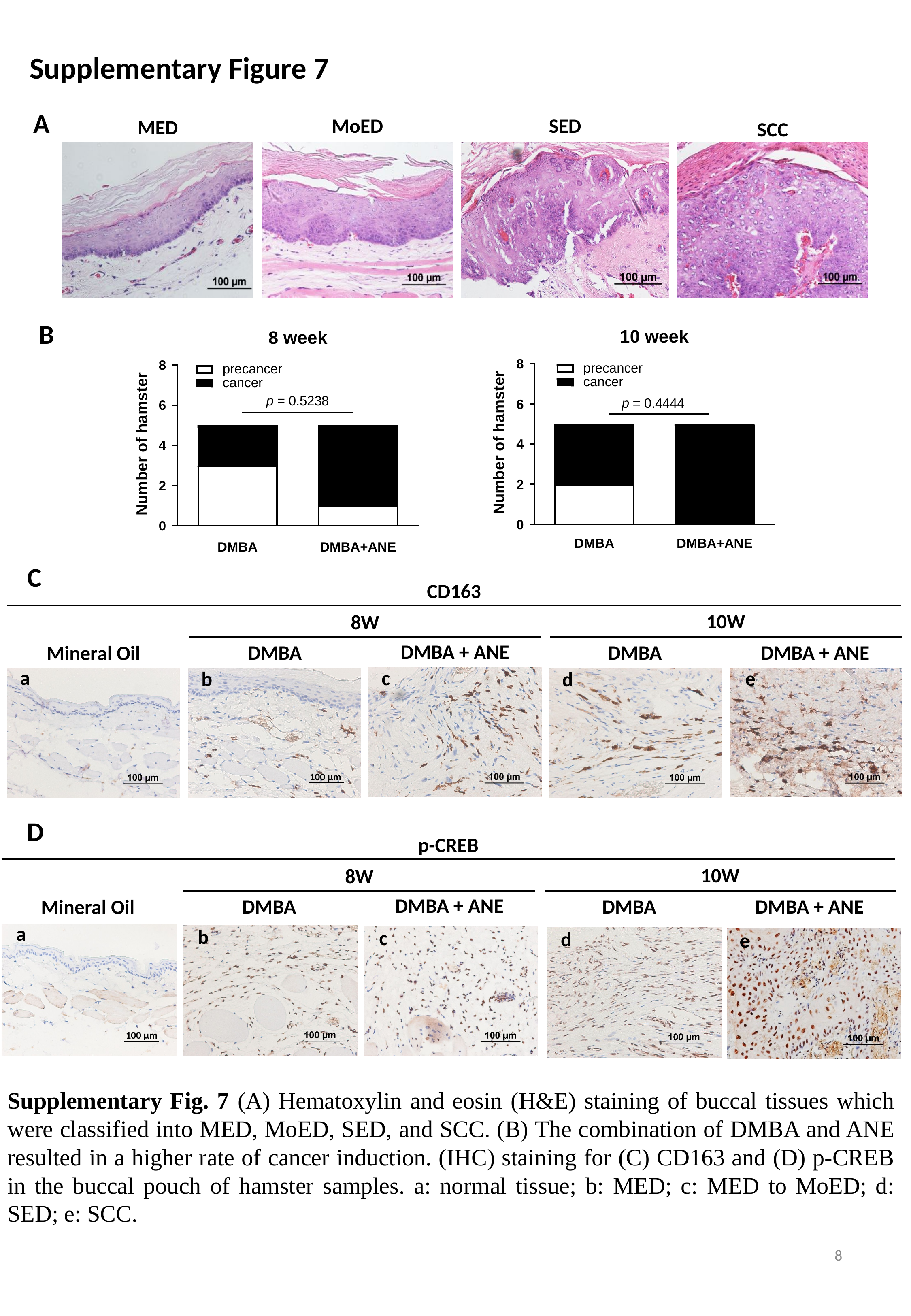

Supplementary Figure 7
A
MoED
SED
MED
SCC
B
1
C
CD163
10W
8W
DMBA + ANE
DMBA
DMBA + ANE
DMBA
Mineral Oil
a
c
e
b
d
100 μm
D
p-CREB
10W
8W
DMBA + ANE
DMBA
DMBA + ANE
DMBA
Mineral Oil
a
b
c
d
e
100 μm
Supplementary Fig. 7 (A) Hematoxylin and eosin (H&E) staining of buccal tissues which were classified into MED, MoED, SED, and SCC. (B) The combination of DMBA and ANE resulted in a higher rate of cancer induction. (IHC) staining for (C) CD163 and (D) p-CREB in the buccal pouch of hamster samples. a: normal tissue; b: MED; c: MED to MoED; d: SED; e: SCC.
8
